# A Systematic Review and Meta-Analysis of Noise Annoyance as a Determinant of Physiological Changes Linked to Disease Promotion

**DOI:** 10.3390/ijerph21070956

**Published:** 2024-07-22

**Authors:** Emily Senerth, Tejanth Pasumarthi, Neha Tangri, Bhavya Abbi, Skye Bickett, James P. McNamee, David S. Michaud, Rebecca L. Morgan

**Affiliations:** 1 Evidence Foundation, 17415 Shelburne Road, Cleveland Heights, OH 44118, USA; emily.senerth@gmail.com (E.S.); pasumart@mcmaster.ca (T.P.); nehatangri2000@gmail.com (N.T.); snbickett@gmail.com (S.B.); rlm180@case.edu (R.L.M.); 2 Faculty of Science, McMaster University, Hamilton, ON L8S 4L8, Canada; bhavyabbi@gmail.com; 3 Health Canada, Environmental and Radiation Health Sciences Directorate, Consumer & Clinical Radiation Protection Bureau, Ottawa, ON KIA ICA, Canada; james.mcnamee@hc-sc.gc.ca; 4 School of Medicine, Case Western Reserve University, Cleveland, OH 44106, USA; 5 Department of Health Research Methods, Evidence and Impact, McMaster University, Hamilton, ON L8S 4L8, Canada

**Keywords:** noise annoyance, biological risk factors, allostatic load, stress, disease, systematic review

## Abstract

This systematic review investigates the certainty of evidence (CoE) regarding noise annoyance as a determinant of biological changes known to contribute to disease development. We searched PubMed MEDLINE, EMBASE, Cochrane Central, and CINAHL for English-language comparative studies conducted on humans of any age from 1 January 1940, to 28 August 2023. Further, studies that provided quantitative data on the relationship between noise annoyance and biomarkers of interest were included. Where possible, random-effects meta-analyses were used to calculate the odds ratios of noise annoyance on biomarkers and biological conditions considered to be risk factors for developing health effects. The risk of bias of individual studies was assessed using the Risk of Bias of Non-randomized Studies of Exposures (ROBINS-E) instrument. The CoE for each outcome was assessed using the Grading of Recommendations Assessment, Development and Evaluation (GRADE) approach. The search identified 23 primary studies reporting on relevant biomarkers. Although some studies and pooled estimates suggest a possible association between noise annoyance and biological measures, the CoE overall is very low due to concerns with the risk of bias, inconsistency, and imprecision in the estimates of effects. In the context of environmental impact assessment, where guidelines aim to mitigate the prevalence of populations experiencing a high level of noise annoyance, our results suggest that such practices should be grounded in the understanding that annoyance is health-relevant because it reflects an undesirable reaction to noise, rather than a precursor to chronic physical health conditions.

## 1. Introduction

Noise has been studied in relation to numerous extra-aural stress-related metabolic effects, sleep disturbance, and cardiovascular disease for several decades, yet there remains a great deal of inconsistency in its association with the evaluated outcomes and the magnitude of association [1,2,3,4,5]. According to the World Health Organization (WHO), as many as 1.6 million healthy life years may be lost each year in urban areas across Europe due to noise exposure [6]. While noise thresholds and guidelines have been implemented to reduce adverse health effects, the mechanisms by which noise impacts human health beyond the auditory system are not entirely known. Nevertheless, the annoyance response has long been considered to fall along the indirect pathway between exposure and adverse outcomes [7].

As the most common reaction to an unwanted noise exposure, noise annoyance is characterized as an adverse reaction to environmental noise. Its manifestation at a sustained and high magnitude can be interpreted as an inability to cope with an exposure, leading to stressor reactions, which may contribute to allostatic load. While the body’s stress responses are essential for survival, sustained stress is associated with chronic activation and dysregulation of stress response systems that in turn are associated with an increased risk of disease [8]. This has been conceptualized as allostatic load—essentially the wear and tear on the body from stressor exposure [9,10]. Allostatic load has typically been operationalized by assessing whether measures that represent our stress response systems or their downstream effects—notably endocrine, inflammatory, cardiovascular, and metabolic—surpass risk thresholds. Understanding how noise annoyance may affect these systems would inform how noise annoyance is interpreted in the context of noise policy in general and environmental impact assessment in particular.

Health Canada provides advice on the potential health impacts of noise exposure in support of the federal environmental impact assessment process [11]. Although other population responses are considered (e.g., sleep disturbance), a central facet of Health Canada’s advice on noise and human health emphasizes predicting the change in prevalence of high noise annoyance experienced by a community exposed to project-related changes in noise. Although there exists a variety of Canadian, U.S., and international criteria and targets for noise mitigation with respect to environmental impact assessment, Health Canada’s use of annoyance is consistent with other provincial jurisdictions and precedents from the U.S. and international standard-setting bodies [12,13]. However, noise annoyance in Health Canada’s federal environmental impact assessment evaluation process is largely rooted in its alignment with the expansive World Health Organization (WHO) definition of health, affirmed in the WHO (2020) Constitution as “a state of complete physical, mental and social well-being and not merely the absence of disease or infirmity” (p.1) rather than the strength of its correlation with established biological risk factors, such as those activated in response to stressor exposure [14].

This systematic review presents a comprehensive overview of the available evidence on the relationship of noise annoyance to biomarkers that precede health effects, including those related to cardiovascular diseases. This review aims to inform researchers and policymakers and to support future research and strategies to manage the potential effects of noise annoyance. The authors acknowledge that sustained noise-induced sleep disturbance has been proposed as a pathway to adverse health; however, this falls outside the scope of the current review. In addition, this review does not consider annoyance as a potential precursor to mental health [15,16]. The results of our analysis would only be applicable to depression, anxiety, or other mental illnesses where the biomarkers evaluated are established as risk factors for such conditions.

## 2. Methods

We conducted a systematic review of the association between noise annoyance and certain biomarkers that may precede adverse health effects. We followed the Preferred Reporting Items for Systematic Reviews and Meta-Analyses (PRISMA) checklist for the development of this review [17]. The protocol is registered in PROSPERO: CRD42023465309.

### 2.1. Literature Search

An information specialist conducted searches in MEDLINE via PubMed, EMBASE, Cochrane Central, and CINAHL from 1 January 1940, through 28 August 2023, for peer-reviewed primary studies reporting on noise annoyance and biomarkers related to cardiovascular diseases, oxidative stress, and diabetes (Supplemental Table S1). The search was restricted to English-language studies. Additionally, we conducted a search of the references from prior systematic reviews on the effect of noise on stress to identify any additional sources of data on biomarkers [1,2,3].

### 2.2. Study Selection

The Population, Exposure, Comparator, and Outcomes (PECO) model was applied. Humans of any age made up the population, noise annoyance was considered the exposure, the degree of annoyance the comparator, and biomarkers of interest constituted the outcome. Studies conducted on humans of any age that provided quantitative data on the relationship between noise annoyance and biomarkers of interest were included. Studies reporting any source of noise, duration of noise annoyance, and any self-reported measure of noise annoyance were eligible. Eligible cardiovascular biomarkers include C-reactive protein (CRP), cortisol, brain-derived neurotrophic factor (BDNF), aldosterone, interleukin-6 (IL-6), tumor necrosis factor-alpha (TNF-α), adrenomedullin, endothelin-1 (ET-1), norepinephrine, epinephrine, troponin, lipid profile (elevated total cholesterol, low-density lipoprotein [LDL] cholesterol, and triglycerides, as well as decreased high-density lipoprotein [HDL] cholesterol), fibrinogen, homocysteine, oxidized low-density lipoprotein (ox-LDL), heart rate variability (HRV), systolic blood pressure, diastolic blood pressure, brain natriuretic peptide (BNP), D-dimer, soluble intercellular adhesion molecule-1 (sICAM-1), myeloperoxidase (MPO), pro-atrial natriuretic peptide, and galectin-3. Eligible oxidative stress biomarkers include reactive oxygen species (ROS), malondialdehyde (MDA), superoxide dismutase (SOD), catalase (CAT), glutathione peroxidase, total antioxidant capacity (TAC), nitric oxide (NO), advanced oxidation protein products (AOPPs), and 8-hydroxy-2′-deoxyguanosine (8-OHdG). Eligible biomarkers related to diabetes include fasting plasma glucose (FPG), glycated hemoglobin (HbA1c), oral glucose tolerance test (OGTT), insulin, C-peptide, adiponectin, high-sensitivity C-reactive protein (hsCRP), and lipid profile. This extensive list was considered based on evidence of their association with the development of stress-related adverse health conditions [10].

Two raters reviewed titles and abstracts independently and in duplicate. Studies that met eligibility criteria during the first stage of screening progressed to full-text review, which was also conducted independently and in duplicate using the screening software program Covidence (Covidence systematic review software, Veritas Health Innovation, Melbourne, Australia. Available at www.covidence.org accessed on 31 May 2024). Discrepancies were resolved through discussion among reviewers; if consensus was not achieved, a third reviewer was involved.

### 2.3. Data Collection

Data extraction was performed by one researcher using a standardized and pilot-tested data extraction form and reviewed by a second reviewer. The following information was collected for each included study: publication information, study design, study population, source of noise, measure of exposure (i.e., noise annoyance for the purpose of this review), measure of outcome, statistical analysis performed, estimates for relevant outcomes, and funding information. Discrepancies were resolved through discussion or consultation with a third reviewer. We abstracted adjusted estimates whenever these were provided, and if multiple estimates were included, we reported both adjusted and unadjusted results in the GRADE summary of findings.

### 2.4. Data Analysis

When appropriate, data were synthesized quantitatively and pooled in a random-effects meta-analysis. We considered the “highly annoyed” (HA) group from each study to be exposed according to the standardization approach described in ISO/TS 15666:2021 [18]. Separate random-effects meta-analyses were conducted for each outcome. If continuous measures were not available, studies with comparable noise annoyance exposure scales were grouped and analyzed in a random-effects meta-analysis using the generic inverse variance method. When pooling binary outcomes, the odds ratios (ORs) were log-transformed. All meta-analyses were performed using the Cochrane Review Manager (version 5.4, The Cochrane Collaboration, London, UK). Heterogeneity between studies was assessed by visual inspection of the forest plots and the I^2^ statistic.

When we were unable to pool studies due to differences in measurement of exposure (annoyance) or outcome, or reporting of outcomes, or when only one study was available for a given outcome, findings were tabulated and described narratively. Due to the limited quantitative outputs, we were unable to conduct dose–response analyses. For this reason, it was not possible to evaluate the evidence for how the magnitude of reported annoyance (e.g., slight, moderately, very, extremely) affected the outcomes considered.

### 2.5. Risk of Bias

Risk of bias assessments were performed using the Risk of Bias Instrument for Non-randomized Studies of Exposures (ROBINS-E) for non-randomized (i.e., observational) studies [19]. ROBINS-E assesses the risk of bias in a study against a well-done target experiment by evaluating the identification and appropriate adjustments of critical confounders. The following confounders were identified as critical for adjustment in the analysis because of their influence on annoyance and/or the outcomes considered: age, sex, noise sensitivity, ethnicity, socioeconomic status (SES), and sleep disturbance. Noise level was not identified as a critical confounder because its putative effects on health are presumed to be mediated through annoyance. It is important to note that the fully adjusted estimate from each of the studies was used. If the original study controlled or adjusted for noise level, that estimate was included in the meta-analysis or reporting of individual study results. Noise level was not identified as a critical confounder for the purposes of risk of bias assessment, but none of the studies adjusted for all other critical confounders, so the addition of noise level would not change any of these judgments. Therefore, the expression of noise annoyance was treated as the “exposure” in the current analysis. Each study was assessed across seven domains for bias resulting from the following: confounding; measurement of the exposure; selection of participants; post-exposure interventions; missing data; measurement of the outcome; and the selection of the reported result. Algorithms for arriving at domain-level judgments are presented in the ROBINS-E manual at https://www.riskofbias.info (accessed on 26 June 2024). In brief, for each domain, reviewers completed signaling questions informing a domain-level judgment of the following: low risk of bias; some concerns; high risk of bias; or very high risk of bias. Low risk of bias means that there was little or no concern about bias with regard to this domain. Some concerns with risk of bias means that there was some concern about bias with regard to this domain, although it was not clear that there was an important risk of bias. High risk of bias means that the study had some important problems in this domain: characteristics of the study give rise to a high risk of bias. Very high risk of bias means that the study was very problematic in this domain: characteristics of the study give rise to a very high risk of bias.

One reviewer performed the initial risk of bias assessment for each study independently, and their judgments were reviewed by a second researcher. All publications or records for a single primary study were considered when making risk of bias judgments. Discrepancies between assessments were resolved by consensus or consultation with a third reviewer. The results from the domain-level risk of bias assessments per study are displayed in a risk of bias matrix (Section 3).

### 2.6. GRADE Evidence Assessment

The overall certainty of the evidence was assessed across each outcome by study design. For this review, the body of evidence for both random clinical trials (RCTs) and non-randomized studies started at a high initial certainty of evidence (CoE). This was possible, as the risk of bias was assessed using ROBINS-E, which assessed risk of bias in comparison to a well-designed target experiment.

Following the GRADE approach, the CoE for each outcome was assessed for concerns with any of the following five domains leading to rating down at least one level: risk of bias, inconsistency, indirectness, imprecision, or publication bias [20]. For non-randomized studies, if there were no concerns beyond risk of bias, then the certainty for the body of evidence could be rated up due to three domains: large or very large magnitude of effect, dose–response gradient, opposing residual confounding. After the assessment of these domains, the body of evidence for an outcome could have a final certainty level of high (⨁⨁⨁⨁), moderate (⨁⨁⨁), low (⨁⨁), or very low (⨁). High certainty means that we are very confident that the true effect lies close to that of the estimate of the effect. Moderate certainty means that we are moderately confident in the estimate of effect; the true effect is likely to be close to the estimate of effect, but possibly substantially different. Low certainty means that our confidence in the effect is limited; the true effect may be substantially different from the estimate of the effect. Lastly, very low certainty means that we have very little confidence in the effect estimate, and the true effect is likely to be substantially different from the estimate of the effect.

One reviewer initially populated the GRADE evidence profiles and assessed the CoE for each outcome of interest. Judgments were reviewed by a second researcher, and disagreements were resolved through discussion. The results of the GRADE assessment are presented comprehensively in an evidence profile.

## 3. Results

### 3.1. Summary of the Literature

**Figure 1:**
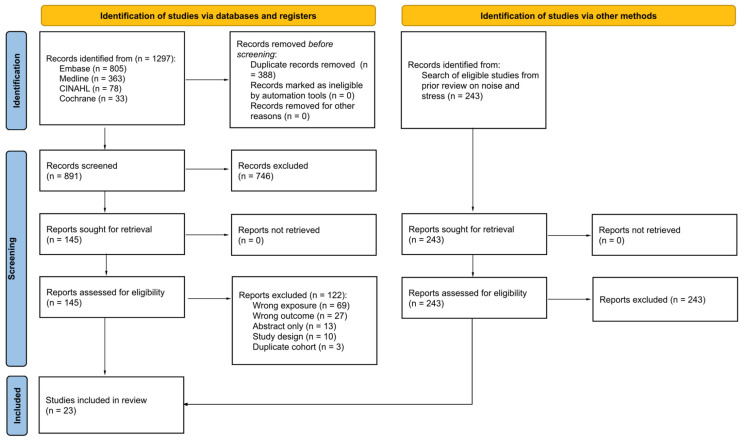
PRISMA flow diagram.

Our search identified 1297 records, of which 23 met eligibility criteria (Figure 1). Most of the studies excluded after retrieval of full texts reported investigating the health effects of noise exposure but did not report on the “exposure” of noise annoyance (Table S2). Studies were also excluded if they reported on long-term health outcomes (e.g., myocardial infarction, ischemic heart disease) rather than biomarkers that are known to fall along the causal pathway of developing cardiovascular diseases from stressor exposure. Conference abstracts and protocols were excluded after we searched for a peer-reviewed publication of the results. Narrative reviews and commentaries were excluded based on their study design.

Our search identified 25 primary research studies that reported on 14 of 48 outcomes of interest (Table 1). Out of 25 eligible studies, 2 (8%) reported the same outcomes from overlapping populations and were ultimately excluded, bringing the total number of included studies to 23. Most of the studies (*n* = 21, 91.3%) were cross-sectional in design and had large samples drawn from the local population (number of study participants ranging from 20 to 14,639). One study (4.3%) was both cross-sectional and longitudinal [21]; one (4.3%) was a cohort study [22]. Additionally, most of the studies (*n* = 21) included only adults in their sample, while two studies were focused exclusively on children and adolescents [23,24].

### 3.2. Measurement of Exposure

All studies used self-reported measures of noise annoyance completed by study participants. Noise annoyance assessment instruments varied across studies from dichotomous (no annoyance, any annoyance) to 12-point scales. The most common measure was an 11-point (0–10) scale using the International Commission on the Biological Effects of Noise (ICBEN) question, “Thinking about the last 12 months, when you are here at home, how much does [type of noise] bother, disturb or annoy you?” (Table 2) [25]. Additional scales used to measure annoyance are described in the results.

### 3.3. Measurement and Analysis of Confounders

One of the studies adjusted for all six of the critical confounders, though their measure of ethnicity was dichotomous (Bulgarian or not). Two studies adjusted for four of the six critical confounders. Eight studies adjusted for three of six critical confounders. Three studies adjusted for two critical confounders. One study adjusted for a single critical confounder. Seven studies failed to adjust for any critical confounders. Despite incomplete adjustment for critical confounders, we conducted a full ROBINS-E assessment for all studies. The results of the risk of bias assessment are presented in Table 3 with the associated risk of bias judgement also defined in Table 3.

### 3.4. Outcomes

The included studies reported on outcome measures for cardiovascular, oxidative stress, and diabetes biomarkers. Cardiovascular biomarker outcomes reported in the literature included blood pressure, lipid profile, atrial fibrillation, arterial stiffness, cortisol, heart rate variability (HRV), and pro-atrial natriuretic peptide (pro-ANP). Two studies reported on catecholamines (adrenaline and noradrenaline) for oxidative stress biomarkers. Reported biomarkers associated with diabetes are glomerular filtration rate (GFR), lipid profile, alpha-amylase, and renal plasma flow (RPF). The summary of findings for the body of evidence for each outcome is presented in Table 4.

#### 3.4.1. Hypertension

Although hypertension is sometimes viewed as a long-term health effect, it is also considered a biological risk factor for cardiovascular and other diseases and was therefore within the scope of the current review. We found ten studies that reported on the impact of noise annoyance on the prevalence of hypertension. Noise annoyance was measured using an 11-point scale [28,29,32,39,40,43], a 3-point scale [41,44], a 10-point scale [31], or a 6-point scale [23].

**Figure 2:**
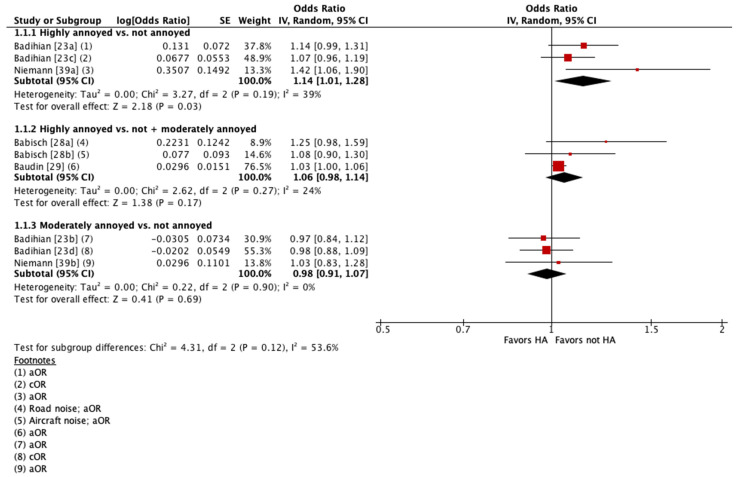
Meta-analysis of hypertension prevalence (all estimates).

**Figure 3:**
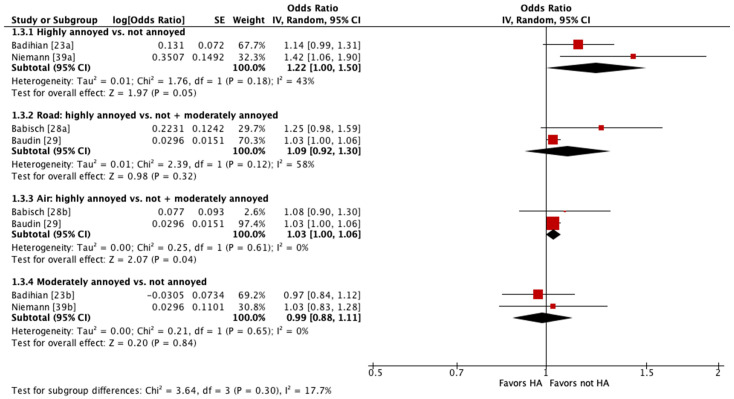
Meta-analysis of hypertension prevalence (adjusted estimates).

Four of these studies were pooled into meta-analyses (Figure 2 and Figure 3) [23,28,29,39]. When pooling adjusted estimates for highly annoyed vs. not annoyed participants, we found that highly annoyed individuals may be at increased risk of hypertension, yet the 95% confidence interval (CI) indicated the association was tenuous (OR 1.22 [95% CI 1.0 to 1.5]). The unadjusted estimate reported by one of the studies pooled showed a weaker effect to no effect of annoyance on hypertension (OR 1.07 [95% CI 0.96 to 1.19]) [23]. When pooling adjusted estimates for highly annoyed vs. all other participants, including both moderately annoyed and not annoyed, we found that the level of annoyance may have little to no effect on hypertension (OR for road noise 1.09 [95% CI 0.92 to 1.30]; OR for aircraft noise 1.03 [95% CI 1.0 to 1.06]). Finally, we pooled adjusted estimates for moderately annoyed vs. not annoyed participants and found that the magnitude of annoyance may have little to no effect on hypertension (OR 0.99 [95% CI 0.88 to 1.11]). The unadjusted estimate reported by one of the studies pooled is consistent with the pooled adjusted results (OR 0.98 [95% CI 0.88 to 1.09]) [23]. The overall certainty in the evidence is very low due to extremely serious concerns with risk of bias (Table 4).

The other six studies could not be pooled because they analyzed noise annoyance as a continuous variable [40,43], did not report the 95% CI for the effect estimate [44], reported prevalence as a percentage difference between groups [41], or did not report any effect estimate and only commented on the statistical significance of the association [31,32].

When comparing the prevalence of hypertension between highly annoyed and not highly annoyed study participants, two cross-sectional studies reported that “no significant effects/relationship” were found; results were not reported quantitatively [31,32]. These studies measured annoyance using an 11-point [32] and a 3-point [31] scale. Four other cross-sectional studies reported that highly annoyed status may be associated with an increased prevalence of hypertension [40,41,43,44]. Petri et al. measured noise annoyance using an 11-point scale and reported an unadjusted OR per 5dB(A) increase in noise of 1.09 (95% CI 0.92–1.11) [40]. Vandasova et al. measured noise annoyance using a 3-point scale and reported an unadjusted OR of 1.5 (no 95% CI reported) [44]. Stosic et al. reported a Spearman R-coefficient for high annoyance measured via a 5-point verbal scale of 0.216 (t = 4.41), and for high annoyance measured via an 11-point numeric scale of 0.211 (t = 4.38) [43]. Pitchika et al. dichotomized the noise annoyance and reported that participants who indicated any annoyance by traffic noise in either their living room or bedroom had a 39% higher prevalence of hypertension (95% CI: 5.9% to 81.2%) compared to participants who were not annoyed in either of the rooms [41]. The overall CoE is very low due to concerns with risk of bias and inconsistency (Table 4).

#### 3.4.2. Blood Pressure

Five cross-sectional studies reported on the impact of noise annoyance on systolic blood pressure (SBP) and diastolic blood pressure (DBP) [26,30,37,38,41]. Three of the studies measured noise annoyance using an 11-point scale [26,30,37,38]. One study measured annoyance using a numerical rating scale from 1 to 10 and then dichotomized responses into “not at all” (1) and “at least some annoyance” (2–10) [41]. One study asked respondents “Do you feel annoyed by that exposure or not?” Noise annoyance was coded as “yes” when the duration of exposure exceeded 50% of working hours [37]. All studies reported a possible association between increasing noise annoyance and increasing DBP.

Dzhambov et al. reported an adjusted SBP change of 0.22 (95% CI—2.82, 3.27) per one interquartile range increase in global noise annoyance [30]. Alimohammadi et al. reported an adjusted Beta estimate for increase in SBP of 3.27 (SD 0.36) with increasing noise annoyance [26]. Michaud et al. reported a geometric mean SBP of 123.02 mmHg (95% CI 119.07 to 127.10) in the highly annoyed group and a geometric mean SBP of 117.99 mmHg (95% CI 116.60 to 119.40) in the low-annoyance group; this difference was not statistically significant [38]. Pitchika et al. reported an adjusted 0.8% mean change in SBP (95% CI -0.6 to 2.1) for the group that reported at least some annoyance in their living room or bedroom compared to the participants who reported no annoyance in either room [41].

Four studies also reported a possible association between increasing noise annoyance and increasing SBP [26,30,38,41]. Dzhambov et al. reported an adjusted DBP change of 0.33 (95% CI—1.56, 2.21) per one interquartile range of increase in global noise annoyance [30]. Alimohammadi et al. reported an adjusted Beta estimate for an increase in DBP of 1.05 (SD 0.27) with increasing noise annoyance [26]. Michaud et al. reported a geometric mean DBP of 76.99 mmHg (95% CI 74.55 to 79.52) in the highly annoyed group and a geometric mean DBP of 73.78 mmHg (95% CI 72.91 to 74.65) in the low-annoyance group; this difference was statistically significant [38]. Pitchika et al. reported an adjusted 0.1% mean change in DBP (95% CI -1.1 to 1.4) for the group that reported at least some annoyance in their living room or bedroom compared to the participants who reported no annoyance in either room [41]. One study reported that noise annoyance may result in an increase or a decrease in SBP (OR 2.1 [95% CI—3.0 to 7.3]) [37]. The overall CoE for both SBP and DBP is very low due to concerns with risk of bias and imprecision (Table 4).

#### 3.4.3. Lipid Profile

One cross-sectional study reported on the impact of noise annoyance on serum lipid profile from a single fasted morning blood sample [21]. Annoyance was measured using a 4-point scale. When comparing highly annoyed males exposed to high noise levels in an occupational setting to those who reported low levels of annoyance in a low-noise setting, the study reported a 15 mg/dL higher mean cholesterol level (95% CI 7.2 to 22.8). When comparing highly annoyed women in a high-noise setting to those who reported low levels of annoyance in a low-noise setting, the study reported a 23 mg/dL higher mean cholesterol level (95% CI 1.5 to 42.9). The CoE is very low due to extremely serious concerns with risk of bias (Table 4). It is also noted that although the authors assessed hearing protection usage (yes/no), they did not report the prevalence of use, nor did they analyze hearing protection as a covariate. Moreover, a potential influence of annoyance alone cannot be disentangled from effects owing to the different settings.

#### 3.4.4. Atrial Fibrillation

One cross-sectional study reported on the impact of noise annoyance on the prevalence of atrial fibrillation (AF) assessed via self-reported medical history and/or documentation of AF on the study electrocardiogram [33]. Annoyance was measured using a 5-point numerical rating scale. They found that study participants who reported “extreme annoyance” had a higher prevalence of AF (23.4%) compared to participants who reported “no annoyance” (14.6%). The certainty in the evidence is very low due to extremely serious concerns with risk of bias (Table 4).

#### 3.4.5. Arterial Stiffness

One cross-sectional study reported on the impact of noise annoyance on arterial stiffness (also known as arterial hypertension [AH]) measured with the carotid–femoral pulse wave velocity and components of blood pressure [42]. Annoyance was measured using a 3-point scale. They reported no association between highly annoyed and moderately annoyed status with AH in all study participants. When the investigators stratified participants into normotensive and hypertensive participants, the normotensive group results indicated a possible positive association between noise annoyance and AH; there was no association in the hypertensive group. The CoE is very low due to extremely serious concerns with risk of bias (Table 4).

#### 3.4.6. Cortisol

Two cross-sectional studies reported on the impact of noise association and cortisol levels [24,38]. Wallas et al. measured annoyance using a 5-point verbal rating scale and determined cortisol concentrations from a morning and evening saliva sample [24]. They found no association between being very annoyed or moderately annoyed and salivary cortisol levels. Michaud et al. measured noise annoyance using an 11-point scale and measured cortisol levels from 3 cm (i.e., ~90-day integrated cortisol) hair samples [38]. In their unadjusted models, they reported a statistically significant difference in hair cortisol levels between highly annoyed participants (geometric mean 169.79 ng/g [95% CI 124.86 to 230.89]) and the not highly annoyed group (geometric mean 127.64 ng/g [95% CI 112.60 to 144.68]). These differences were no longer significant in their fully adjusted models. The CoE is very low due to extremely serious concerns with risk of bias (Table 4).

#### 3.4.7. Heart Rate Variability (HRV)

Two cross-sectional studies reported on the impact of noise annoyance on heart rate variability and found “no significant effects/difference” based on annoyance level; results were not reported quantitatively [32,35]. Gallasch et al. measured HRV using a Task Force Monitor (TFM), which uses a standard three-electrode system for ECG, a four-electrode system for ICG, and two inflatable finger cuffs in combination with a standard arm cuff [32]. Huang et al. measured HRV using heart rate recording [35]. Annoyance was measured using an 11-point interval scale [32] and an 11-point visual analog scale [35]. The overall CoE is very low due to extremely serious concerns with risk of bias (Table 4).

#### 3.4.8. Pro-Atrial Natriuretic Peptide (Pro-ANP)

One cross-sectional study reported on the impact of noise annoyance on pro-ANP levels determined from a single overnight 8-hour fasted venous blood sample [34]. Annoyance was measured using a 5-point numerical rating scale. They found an increase in pro-ANP with each point increase in annoyance of 0.013 pmol/L (95% CI 0.0041 to 0.021). Increasing daytime noise annoyance was associated with an increase of 0.014 pmol/L (95% CI 0.0057 to 0.023) per point; increasing nighttime annoyance was associated with an increase of 0.011 pmol/L (95% CI 0.0019 to 0.020) per point. The CoE is very low due to extremely serious concerns with risk of bias (Table 4).

#### 3.4.9. Catecholamines (Adrenaline and Noradrenaline)

One cross-sectional study [27] and one cohort study [22] reported on the impact of noise annoyance on overnight urinary adrenaline and noradrenaline [27] and prior to and between arithmetic testing during noise [22]. Annoyance was measured using a 5-point numerical rating scale [27] and a verbal category scale with no information reported on the number of categories [22]. When comparing “highly disturbed” individuals with “less disturbed” individuals, Babisch et al. found that increasing disturbance in communication (regression coefficient 1.0 µg/g creatinine), sleep (regression coefficient 5.57 µg/g creatinine), and relaxation (regression coefficient 0.59 µg/g creatinine) may lead to increasing levels of catecholamines [27].

When comparing “quite annoyed” to “not especially annoyed” participants, Arvidsson and Lindvall found that levels of both adrenaline (mean 12.5 ng/min [SD 6.6] vs. mean 17.6 ng/min [SD 7.5]) and noradrenaline (mean 29.3 ng/min [SD 12.1 vs. mean 36.3 ng/min [SD 10.7]) were lower in the “quite annoyed” group. The CoE is very low due to extremely serious concerns with risk of bias (Table 4) [22].

#### 3.4.10. Glomerular Filtration Rate (GFR)

Two cross-sectional studies reported on the impact of noise annoyance on GFR as an estimate of overall kidney function, calculated according to accepted epidemiologic practices [30,36]. Annoyance was measured using an 11-point numerical rating scale [30] and a 7-grade Likert scale [36]. Dzhambov et al. found that for each interquartile range increase in global noise annoyance, eGFR increased by 0.20 mL/min/1.73 m^2^ (95% CI—3.97 to 4.36) [30]. Kannenkeril et al. reported that annoyed participants (mean 133 mL/min [SD 12]) had slightly lower levels of GFR compared to those who were not annoyed (mean 138 mL/min [SD 15]) [36]. The CoE is very low due to concerns with risk of bias and imprecision (Table 4).

#### 3.4.11. Alpha-Amylase

One cross-sectional study reported on the impact of noise annoyance on salivary alpha-amylase levels collected at rest and after road, rail, and calm conditions [32]. Annoyance was measured using an 11-point interval scale. They found “no significant effects”; however, they did not report findings quantitatively. The CoE is very low due to concerns with risk of bias and imprecision (Table 4).

#### 3.4.12. Renal Plasma Flow (RPF)

One cross-sectional study reported on the impact of noise annoyance on RPF assessed using the constant-infusion input clearance technique with an infusion of para-aminohippuric acid [36]. Annoyance was measured using a 7-grade Likert scale. They found that noise-annoyed individuals showed lower RPF (mean 599 ± 106 vs. mean 663 ± 124 mL/min, *p* = 0.009), lower renal blood flow (mean 1068 ± 203 vs. mean 1172 ± 225 mL/min, *p* = 0.047), higher filtration fraction (mean 22.7 ± 3.3 vs. mean 21.3 ± 3.0, *p* = 0.012), higher renal vascular resistance (mean 88.9 ± 25.6 vs. mean 75.8 ± 22.9 mm Hg/[mL/min], *p* = 0.002), and higher resistance of afferent arteriole (mean 2439.5 ± 1253.4 vs. mean 1849.9 ± 1242.0 dyn s^−1^ cm^−5^, *p* = 0.001) compared to non-noise-annoyed individuals. The certainty in the evidence is very low due to concerns with risk of bias and imprecision (Table 4).

## 4. Discussion

A sustained high magnitude of noise annoyance is clearly an undesirable reaction to noise at both the individual and the population level. Yet, the extent to which long-term high noise annoyance may impact one’s health is uncertain, and its relevance to environmental impact assessment would be enhanced if it was also found to contribute to physiological effects that are known to be involved in the development of diseases, particularly those that have been attributed to noise [15]. As an example, on a Canadian national survey on transportation noise, highly annoyed respondents were more likely to report that their annoyance negatively impacted their health compared to their less annoyed counterparts; however, no relationship was observed between the magnitude of noise annoyance and self-reported overall health status [45]. This observation suggests that when weighed against myriad factors that influence health, from diet to exercise and genetic predisposition, noise annoyance, even at high magnitudes, may have a diminutive (if any) contribution to overall health status.

The suggested mechanistic framework that has been proposed is that annoyance is a psychological state that represents a degree of mental distress toward (in this case) noise [8,46]. In greater magnitudes of severity, chronic annoyance may reflect an inability to cope with noise. In this scenario, it would be reasonable to suspect that chronic high annoyance with noise has the potential to increase one’s allostatic load by constantly requiring that one adapts to the noise. In susceptible individuals, this could potentially promote disease [9,10]. Although the theoretical connection between noise annoyance and health seems intuitive, the onset of pathophysiology necessitates deviations in biological systems persisting at levels outside the normal physiological range.

We have identified a comprehensive body of evidence reporting on the effect of noise annoyance on biomarkers that precede health effects that have been studied in relation to noise. These outcomes included but were not limited to cardiovascular disease and diabetes. These results contribute to the investigation of mechanisms by which noise exposure may affect human health outcomes unrelated to noise-induced hearing impairment. However, the CoE of a relationship between noise annoyance and the various biomarkers evaluated is very low due to concerns with risk of bias, inconsistency, and imprecision.

The largest body of evidence reported on the outcome of hypertension prevalence, as informed by blood pressure measurements. While only a portion of the studies (*n* = 4) could be pooled, this analysis suggests a possible trend of increasing hypertension among highly annoyed groups when compared with not annoyed or moderately and not annoyed groups. The results were not suggestive of an association when moderate annoyance was compared with no annoyance, which does support the relative emphasis placed on “highly annoyed” in guidance [15]. These results are consistent with the six studies that could not be pooled in the analysis. Studies also reported on systolic and diastolic blood pressure separately, with heterogeneity in their estimates of the effect of noise annoyance on both measures of blood pressure. Furthermore, while some, albeit weak, evidence exists for an association between high noise annoyance and hypertension, the cross-sectional nature of these studies does not permit conclusions about causality.

Other studies reported possible trends of increased mean cholesterol levels, increased prevalence of AF, increased arterial stiffness in normotensive participants, increased hair cortisol levels, increased pro-ANP, and decreased renal plasma flow in highly annoyed groups when compared to not highly annoyed groups. The results for arterial stiffness in hypertensive participants, saliva cortisol, heart rate variability, and catecholamines (adrenaline and noradrenaline) suggest that high annoyance may have no effect on these outcomes.

### Limitations

This review employed rigorous methods to search for available literature, select relevant studies, collect and analyze data, assess the risk of bias, and assess the overall CoE for each outcome. However, we did not consider biomarkers that may underscore mental health endpoints and did not look at other environmental annoyances like odor, visual, or dust annoyance. Most of the studies (*n* = 21) in our sample are cross-sectional, with concerns about confounding and measurement of the exposure (noise annoyance). Our overall certainty is very low due to additional concerns with inconsistency between studies and imprecision due to small sample sizes for some outcomes. When considered in their totality, the results do not provide robust support for interpreting high noise annoyance as a determinant of changes in physiology that lead to disease. Future research in this area may prove otherwise, especially in regard to hypertension; however, there needs to be a concerted effort to reduce the heterogeneity in research designs. The analysis shows that there is substantial variation across studies, which undermines the ability to subject the collective body of research to meta-analysis.

## 5. Conclusion

In the context of environmental impact assessment guidelines that aim to mitigate the prevalence of populations experiencing a high level of noise annoyance, the current state of the scientific literature indicates that such practices should be grounded in the understanding that long-term high noise annoyance is health-relevant because it reflects an undesirable reaction to noise that may affect general well-being, rather than a precursor to chronic physical health conditions.

## Figures and Tables

**Table 1:** Summary characteristics of eligible studies.

	Studies *n* (%)
Total studies	23 (100)
Study design	
Cross-sectional	21 (91.3)
Cohort	1 (4.3)
Cross-sectional and longitudinal	1 (4.3)
Geographic location	
Europe	19 (82.6)
Asia	3 (13)
North America	1 (4.3)
Population	
Children and adolescents (<18 years)	2 (8.7)
Adults and seniors (18–80 years)	21 (91.3)
Noise annoyance measure (all self-reported)	
Dichotomous (no annoyance, any annoyance)	2 (8.7)
3-point scale	3 (13)
4-point scale	1 (4.3)
5-point scale	4 (17.4)
6-point scale	1 (4.3)
7-point scale	1 (4.3)
11-point scale	10 (43.5)
Unspecified scale	1 (4.3)
Outcomes *	
Cardiovascular biomarkers	
Hypertension	10 (43.5)
Systolic blood pressure/diastolic blood pressure	5 (21.7)
Lipid profile	1 (4.3)
Atrial fibrillation	1 (4.3)
Arterial stiffness	1 (4.3)
Heart rate variability (HRV)	2 (8.7)
Cortisol	2 (8.7)
Pro-atrial natriuretic peptide (pro-ANP)	1 (4.3)
ECG/ICG parameters	0
C-reactive protein (CRP)	0
Brain-derived neurotrophic factor (BDNF)	0
Aldosterone	0
Interleukin-6 (IL-6)	0
Tumor necrosis factor-alpha (TNF-α)	0
Adrenomedullin	0
Endothelin-1 (ET-1)	0
Norepinephrine	0
Epinephrine	0
Troponin	0
Fibrinogen	0
Homocysteine	0
Oxidized low-density lipoprotein (ox-LDL)	0
Brain natriuretic peptide (BNP)	0
D-dimer	0
Soluble intercellular adhesion molecule-1 (sICAM-1)	0
Myeloperoxidase (MPO)	0
Galectin-3	0
Oxidative stress biomarkers	
Catecholamines (adrenaline and noradrenaline)	2 (8.7)
Reactive oxygen species (ROS)	0
Malondialdehyde (MDA)	0
Superoxide dismutase (SOD)	0
Catalase (CAT)	0
Glutathione peroxidase	0
Total antioxidant capacity (TAC)	0
Nitric oxide (NO)	0
Advanced oxidation protein products (AOPPs)	0
8-hydroxy-2′-deoxyguanosine (8-OHdG)	0
Diabetes biomarkers	
Glomerular filtration rate (GFR)	2 (8.7)
Lipid profile	1 (4.3)
Alpha-amylase	1 (4.3)
Renal plasma flow (RPF)	1 (4.3)
Creatinine	0
Fasting plasma glucose (FPG)	0
Glycated hemoglobin (HbA1c)	0
Oral glucose tolerance test (OGTT)	0
Insulin	0
C-peptide	0
Adiponectin	0
High-sensitivity C-reactive protein (hsCRP)	0

* A single study may report on more than one biomarker of interest.

**Table 2:** Detailed characteristics of eligible studies.

Study ID	Study Design	Country	Setting and Noise Type	Population N/Age/Sex	Measure of Noise Annoyance	Outcome Measure(s)	Funding Source(s)
Alimohammadi [26]	Cross-sectional	Iran	Automotive industry Avg. 15 years occupational noise	250/mean 36.19 years (±3.75)/NR	NRS 0–1; larger number = more annoyed	BP measured with ALPK2 mercury pressure gauge	Iran University of Medical Sciences
Arvidsson [22]	Cohort	Sweden	University laboratory 15-min (×4) road traffic noise	100/23–30 years/0% female	Verbal category scale	Catecholamines (adrenaline and noradrenaline) measured in urine	Swedish Environmental Protection Board
Babisch [27]	Cross-sectional	Germany	City of Berlin Avg. 8.4 years road traffic noise	195/30–45 years/100% female	NRS 1–5; not at all, little, moderate, much, and very much	Catecholamines (adrenaline and noradrenaline) measured in urine Creatinine measured photometrically using an Eppendorf 1101M photometer	NR
Babisch [28] (HYENA)	Cross-sectional	England, Germany, Sweden, Netherlands, Italy, Greece	Cities near 6 major European airports Min. 5 years aircraft noise	4861/45–70 years/51% female	NRS 0-10; ICBEN question “Thinking about the last 12 months, when you are here at home, how much does aircraft noise bother, disturb or annoy you?”	BP	European Commission
Badihian [23] (CASPIAN-V)	Cross-sectional	Iran	Rural and urban living areas History of exposure unspecified; no assessment of noise exposure	14,274/7–18 years/49% female	NRS 10–60; participants were asked to state how much they agreed with 10 written sentences describing different feelings in response to various types of noise, these sentences could be scored from 1 (completely disagree) to 6 (absolutely agree) based on a Likert scale	BP measured with standard non-automated mercury sphygmomanometers	None
Baudin [29] (HYENA + DEBATS)	Cross-sectional	England, Germany, Sweden, Netherlands, Italy, Greece, France	Cities near 10 major European airports Min. 5 years aircraft noise both HYENA and DEBATS	5886/45–70 years and >18 years/NR	NRS 0–10; ICBEN question “Thinking about the last 12 months, when you are here at home, how much does aircraft noise bother, disturb or annoy you?”	BP measured using automated BP instruments	European Commission, French Ministry of Health, the French Ministry of the Environment, French Civil Aviation Authority, Airport Pollution Control Authority, European and International Affairs Department (DAEI) of FSTTAR (French Institute of Science and Technology for Transport, Development and Networks)
Dzhambov [30]	Cross-sectional	Bulgaria	3 tertiary hospitals in the city of Plovdiv History of exposure unspecified; Avg. 31 years living in dwelling; industrial, neighborhood, building, and apartment noise (general) and traffic noise	217/>18 years/52.1% female	NRS 0–10	BP measured using a calibrated aneroid sphygmomanometer with standard cuff-size eGFR calculated according to the CKD-EPI with serum creatinine analyzed using enzymatic creatinine assay and biochemical analyzer	None
Fyhri [31]	Cross-sectional	Sweden	City of Oslo History of exposure to road traffic noise not reported	1842/mean 42 years/54% female	Participants were first asked if they heard noise from a certain source when staying indoors (at home); then, they were asked “Is this noise highly, somewhat or not annoying?”	BP self-reported	NR
Gallasch [32]	Cross-sectional	Austria	Participants were recruited from a database of individuals who performed a separate traffic noise annoyance test 20min (x3) exposure to recorded road and rail noise	59/20–58 years/51% female	Standardized traffic noise annoyance test based on an 11-graded interval scale; participants were assigned to either a high- or low-annoyance subgroup	ECG, ICG, and continuous BP, all recorded with the Task Force Monitor (TFM); this monitor uses a standard 3-electrode system for ECG, a 4-electrode system for ICG, and two inflatable finger cuffs in combination with a standard arm cuff for BP Alpha-amylase collected in saliva	Hygiene Fund of the Medical University of Graz
Hahad [33] (Gutenberg Health Study)	Cross-sectional	Germany	General population Time at current residence 15–17 years. Industrial, rail, neighborhood, road traffic, and aircraft noise	14,639/35–74 years/52% female	5-point NRS from “not” to “extremely” participants were asked to rate “how annoyed have you been in the past years by noise resulting from road traffic, aircraft, railways, industrial/ construction and neighborhood?”	AF measured by previous diagnosis and/or documentation of AF by ECG	Government of Rhineland-Palatinate (“Stiftung Rheinland- Pfalz für Innovation”, contract AZ 961-386261/ 733), the research programs “Wissen schafft Zukunft” and “Center for Translational Vascular Biology (CTVB)” of the Johannes Gutenberg University of Mainz, and its contract with Boehringer Ingelheim and PHILIPS Medical Systems, including an unrestricted grant for the GHS and by the Foundation Heart of Mainz. T.M., T.G., and P.W. are PIs of the DZHK Partner Site Rhine-Main, Mainz, Germany
Hahad [34] (Gutenberg Health Study)	Cross-sectional	Germany	General population; sample was drawn randomly from the local registry in the city of Mainz and the district of Mainz-Bingen Avg. years at current residence 20 years. Aircraft, road traffic, neighborhood, industrial/construction noise.	4826/35–74 years/50% female	5-point NRS from “not” to “extremely” participants were asked to rate “how annoyed have you been in the past years by noise resulting from road traffic, aircraft, railways, industrial/ construction and neighborhood?”	Midregional pro-atrial natriuretic peptide (MR-pro-ANP) in venous blood determined using a commercially available assay Arterial hypertension defined as systolic BP ≥ 140 mmHg or diastolic BP ≥ 90 mmHg at rest, or by intake of any antihypertensive drugs within the last 2 weeks, or arterial hypertension diagnosed by a physician Dyslipidemia defined as at least one of the following: physician diagnosis of dyslipidemia, low-density lipoprotein cholesterol/ high-density lipoprotein cholesterol ratio > 3.5, or triglycerides ≥ 150 mg/dL	Government of Rhineland-Palatinate (“Stiftung Rheinland- Pfalz für Innovation”, contract AZ 961-386261/ 733), the research programs “Wissen schafft Zukunft” and “Center for Translational Vascular Biology (CTVB)” of the Johannes Gutenberg University of Mainz, and its contract with Boehringer Ingelheim and PHILIPS Medical Systems, including an unrestricted grant for the GHS and by the Foundation Heart of Mainz. T.M., T.G., and P.W. are PIs of the DZHK Partner Site Rhine-Main, Mainz, Germany
Huang [35]	Cross-sectional	Netherlands	Dept of Otorhinolaryngology and Neurology/Clinical Neurophysiology of OLVG West History of exposure and source(s) of exposure not specified	20/median 42 years (IQR: 30.5–57.5)/65% female	VAS 0–10; not annoying, a bit annoying, moderately annoying, quite annoying, extremely annoying	HR variability measured via HR recording	None
Kannenkeril [36]	Cross-sectional	Germany	Clinical Research Unit of the Dept of Nephrology and Hypertension, University of Erlangen, Germany History of exposure to noise in everyday life unspecified	113/NR/0% female	Subjective annoyance due to noise in everyday life on a 7-grade Likert scale	Renal hemodynamics—GFR and RPF assessed using the constant-infusion input clearance technique with infusion of inulin	None
Lercher [37]	Cross-sectional	Austria	Demographically stable rural community Occupational noise; history of exposure duration evaluated in questionnaire but not reported	174/25–64 years/58% female	Respondents were asked “Do you feel annoyed by that exposure or not?” Noise annoyance was coded as “yes” when the duration of exposure exceeded 50% of the working hours	BP measured twice in a sitting position on the right arm	Grant from the Max Kade Foundation
Melamed [21] (CORDIS)	Cross-sectional and longitudinal	Israel	Workplaces with exposure to high industrial noise Mean exposure to various occupational sources of noise ranged from 5.9 years to 16.3 years	3277/20–64 years/29% female	6 4-point items; (1) “Does the noise disturb you?” (1 = not at all, 4 = very much); (2) “How do you feel about the noise level here?” (1 = not annoyed at all, 4 = very annoyed); (3) “Does the noise make you feel so bad that you feel you cannot carry on working? (1 = not at all, 4 = very much so); (4) “Does the noise prevent you from paying attention to your work?” (1 = not at all, 4 = very much); (5) “Does the noise interfere with your communicating with other workers (talk, conversation)?” (1 = not at all, 4 = very much), and (6) “Would your general feeling in the workplace improve without the noise?” (1 = they would not change, 4 = they would improve a lot)	Serum lipid/lipoprotein levels measured in venous blood	Committee for Preventive Action and Research in Occupational Health, The Ministry of Labor and Social Affairs, Jerusalem, Israel
Michaud [38]	Cross-sectional	Canada	Residential areas in southwestern Ontario (ON) and Prince Edward Island (PEI) within the vicinity of wind turbine installations History of exposure (hearing) wind turbine noise analyzed as <1yr vs. ≥1yr	1238/18–79 years/51% female	NRS 0–10; ICBEN question “Thinking about the last 12 months, when you are here at home, how much does traffic/neighborhood/aircraft noise bother, disturb or annoy you?”	Hair cortisol concentrations Systolic BP and diastolic BP measured via automated oscillometric device	Health Canada
Niemann [39] (LARES)	Cross-sectional	France, Germany, Slovakia, Hungary, Spain, Switzerland, Italy, Lithuania	Residential neighborhoods in 8 European cities History of exposure to road traffic and general neighborhood noise not reported	8539/NR/NR	NRS 0–10; ICBEN question “Thinking about the last 12 months, when you are here at home, how much does traffic/neighborhood/aircraft noise bother, disturb or annoy you?”	BP via physician diagnosis	None
Petri [40]	Cross-sectional	Italy	City of Pisa Min. 5yr exposure to transportation noise and recreational noise	517/37–72 years/mean 57.3 years (SD: 8.7)/55.9% female	11-point scale for each source on a list of 10	Systolic BP and diastolic BP measured at home after at least five minutes of rest in a seated position keeping both feet on the ground using an automatic Omron M6 Comfort model	None
Pitchika [41]	Cross-sectional	Germany	City of Augsburg and its two adjacent counties Augsburg and Aichach- Friedberg Analysis restricted to >10 years at current residence, frequency of exposure to road traffic reported as never, seldom, often, or always	2552/31–72 years/mean 52 years/52% female	NRS 1–10; 1, not at all, to 10, intolerable; dichotomized into “not at all” and “at least some annoyance”	BP measured by readings SBP ≥ 140 mm Hg or DBP ≥ 90 mm Hg or self-reported physician-diagnosed hypertension or use of antihypertensive medication in conjunction with self-reported physician-diagnosed hypertension	European Community’s Seventh Framework program (FP7/2007-2011) under grant agreement number: 211250; Helmholtz Zentrum München, German Research Center for Environmental Health (GmbH), which is funded by the German Federal Ministry of Education, Science, Research and Technology and by the State of Bavaria
Rojek [42]	Cross-sectional	Poland	Suburban area of Kraków Min. 3 years exposure to aircraft noise	201/40–65 years/68% female	Subjective noise annoyance measured with a standardized questionnaire; none, moderate, high	BP measured in office twice in the nondominant arm after 10 min of rest using the Omron M5-I device Arterial stiffness measured by the carotid–femoral PWV and components of central BPs using SphygmoCor EM4C device Functional and structural parameters of the left ventricle and left atrium	Jagiellonian University Medical College (grant number K/ZDS/005566)
Stošić [43]	Cross-sectional	Serbia	6 locations in Niš; 3 busy streets and 3 side streets with lower traffic intensity Min. 1 year exposure to road traffic noise	388/18–80 years/57.4% female	NRS 0–10; from 0, not at all, to 10, extremely	BP measured by diagnosis of arterial hypertension by a physician	NR
Vandasova [44]	Cross-sectional	Czech Republic	Urban localities with different noise levels Avg. 22+ years living in area with exposure to road traffic and neighborhood noise sources	5354/30–80 years /58.4% female	Categorical scale 1–3 for each of 12 noise variables; 1, not at all; 2, moderately; and 3, strongly annoyed	BP measured by self-reported hypertension	None
Wallas [24]	Cross-sectional	Sweden	Stockholm County	705/16 years/NR	Verbal rating scale with 5 alternatives; not at all, slightly, moderately, very, and extremely	Cortisol measured in saliva collected by sterile rolls placed in labeled tubes and sent to the laboratory via mail	Swedish Research Council for Health, Working Life and Welfare

AF = atrial fibrillation; BP = blood pressure; CKD-EPI = Chronic Kidney Disease Epidemiology Collaboration equation; ECG = electrocardiogram; GFR = glomerular filtration rate; HR = heart rate; ICBEN = International Commission on the Biological Effects of Noise; ICG = impedance cardiography; NR = not reported; NRS = numerical rating scale; PWV = pulse wave velocity; RPF = renal plasma flow; VAS = visual analog scale.

**Table 3:** Risk of bias assessment for eligible studies.

Study ID	Confounding	Exposure Measurement	Selection	Post-Exposure Intervention	Missing Data	Outcome Measurement	Reported Results
Alimohammadi [26]	Very high risk	Very high risk	Some concerns	Low risk	Low risk	Low risk	Low risk
Arvidsson [22]	Very high risk	Very high risk	Low risk	Low risk	Low risk	Low risk	Low risk
Babisch [27]	Very high risk	Very high risk	Some concerns	Low risk	Low risk	Low risk	Low risk
Babisch [28]	Very high risk	High risk	Some concerns	Low risk	Low risk	Low risk	Low risk
(HYENA)							
Badihian [23]	Very high risk	High risk	Some concerns	Low risk	Low risk	Low risk	Low risk
(CASPIAN-V)							
Baudin [29]	Very high risk	High risk	Some concerns	Low risk	Low risk	Low risk	Low risk
(HYENA + DEBATS)							
Dzhambov [30]	Some concerns	High risk	Low risk	Low risk	Low risk	Low risk	Low risk
Fyhri [31]	Very high risk	Very high risk	Low risk	Low risk	Low risk	Low risk	Low risk
Gallasch [32]	Very high risk	High risk	Low risk	Low risk	Low risk	Low risk	Low risk
Hahad [33]	Very high risk	High risk	Some concerns	Low risk	Low risk	Low risk	Low risk
(Gutenberg Health Study)							
Hahad [34]	Very high risk	High risk	Some concerns	Low risk	Low risk	Low risk	Low risk
(Gutenberg Health Study)							
Huang [35]	Very high risk	High risk	Low risk	Low risk	Low risk	Low risk	Low risk
Kannenkeril [36]	Very high risk	High risk	Some concerns	Low risk	Low risk	Low risk	Low risk
Lercher [37]	Very high risk	Very high risk	Some concerns	Low risk	Low risk	Low risk	Low risk
Melamed [21]	Very high risk	High risk	Some concerns	Low risk	Low risk	Low risk	Low risk
(CORDIS)							
Michaud [38]	Very high risk	High risk	Some concerns	Low risk	Low risk	Low risk	Low risk
Niemann [39]	Very high risk	High risk	Some concerns	Low risk	Low risk	Low risk	Low risk
(LARES)							
Petri [40]	Very high risk	Very high risk	Some concerns	Low risk	Low risk	Low risk	Low risk
Pitchika [41]	Very high risk	High risk	Some concerns	Low risk	Low risk	Low risk	Low risk
Rojek [42]	Very high risk	Very high risk	Some concerns	Low risk	Low risk	Low risk	Low risk
Stošić [43]	Very high risk	High risk	Some concerns	Low risk	Low risk	Low risk	Low risk
Vandasova [44]	Very high risk	Very high risk	Some concerns	Low risk	Low risk	Low risk	Low risk
Wallas [24]	Very high risk	Very high risk	Some concerns	Low risk	Low risk	Low risk	Low risk
**Interpretation of risk of bias judgement.**							
**Judgment**		**Interpretation**					
Low risk		There is little or no concern about bias with regard to this domain					
Some concerns		There is some concern about bias with regard to this domain, although it is not clear that there is an important risk of bias					
High risk		The study has some important problems in this domain: characteristics of the study give rise to a high risk of bias					
Very high risk		The study is very problematic in this domain: characteristics of the study give rise to a very high risk of bias					

**Table 4:** Evidence profile for noise annoyance and biomarkers of diabetes, cardiovascular disease, and oxidative stress.

Certainty Assessment							Impact	Certainty	Importance
No. of Studies	Study Design	Risk of Bias	Inconsistency	Indirectness	Imprecision	Other Considerations			
**Hypertension—single studies (assessed with diagnosis or study measurement of blood pressure)**									
6	non-randomized studies	extremely serious ^a^	serious ^b^	not serious	not serious	none	**Petri [40]:** (*n* = 515) Risk of hypertension among highly annoyed participants vs. not highly annoyed unadjusted OR per 5db(A) increase in noise: 1.09 (95% CI 0.92 to 1.11) **Vandasova [44]:** (*n* = 3592) Risk of hypertension among highly annoyed participants vs. not highly annoyed unadjusted OR: 1.5 (no 95% CI reported) **Stošić [43]:** (*n* = 911) Spearman R-coefficient for highly annoyed (verbal scale): 0.216 (t = 4.41); Spearmen R-coefficient for highly annoyed (numeric scale): 0.211 (t = 4.38) **Pitchika [41]:** (*n* = 2552) 39% (95% CI: 5.9% to 81.2%) higher prevalence of hypertension in participants who were annoyed by traffic noise in either their living room or bedroom vs. participants who were not annoyed in either of the rooms Two studies (**Gallasch [32]** [*n* = 59], **Fyhri [31]** [*n* = 1842]) reported no association between noise annoyance and hypertension; results were not reported quantitatively	⨁◯◯◯ Very low	CRITICAL
**Hypertension—pooled estimates (assessed with diagnosis or study measurement of blood pressure)**									
4	non-randomized studies	extremely serious ^a^	not serious	not serious	not serious	none	**Highly annoyed vs. not annoyed:** OR 1.22 (95% CI 1.0 to 1.5) **Highly annoyed vs. all other participants (moderately annoyed + not annoyed):** OR for road noise 1.09 (95% CI 0.92 to 1.30) OR for aircraft noise 1.03 (95% CI 1.0 to 1.06) **Moderately annoyed vs. not annoyed:** OR 0.99 (95% CI 0.88 to 1.11)	⨁◯◯◯ Very low	CRITICAL
**Blood pressure (assessed with study measurement of blood pressure)**									
4	non-randomized studies	very serious ^c,d^	not serious	not serious	serious ^e^	none	**Dzhambov [30]:** (*n* = 217) Adjusted SBP change 0.22 (95% CI 2.82 to 3.27); adjusted DBP change 0.33 (95% CI 1.56 to 2.21) per one interquartile range increase in global noise annoyance **Pitchika [41]:** (*n* = 2552) Adjusted mean % change for participants annoyed by traffic noise in either their living room or bedroom vs. those not annoyed in either room SBP 0.8% (95% CI −0.6 to 2.1) DBP 0.1% (95% CI −1.1 to 1.4) **Alimohammadi [26]:** (*n* = 250) Adjusted Beta estimate for increase in SBP and DBP with increasing noise annoyance SBP: 3.27 (SD 0.36) DBP: 1.05 (SD 0.27) **Michaud [38]:** (*n* = 1238) Univariate geometric mean (95% CI) estimate of SBP and DBP SBP high annoyance: 123.02 mmHg (95% CI 119.07 to 127.10) SBP low annoyance: 117.99 mmHg (95% CI 116.60 to 119.40) SBP high annoyance: 76.99 mmHg (95% CI 74.55 to 79.52) SBP low annoyance: 73.78 mmHg (95% CI 72.91 to 74.65) **Lercher [37]:** (*n* = 174) Adjusted mean difference for annoyed vs. not annoyed SBP: 2.1 (95% CI -3.0 to 7.3) DBP: 3.5 (95% CI 0.4 to 7.4)	⨁◯◯◯ Very low	CRITICAL
**Lipid profile (assessed with serum cholesterol)**									
1	non-randomized studies	extremely serious ^a^	not serious	not serious	not serious	none	One study (**Melamed [21]** [*n* = 3277]) showed that serum cholesterol levels covaried with reported noise annoyance, and overall levels were higher in participants exposed to high noise levels. High-noise-annoyed workers (men) exposed to high noise levels had a 15 mg/dL higher mean cholesterol level (95% CI 7.2 to 22.8) vs. low-noise-annoyed workers exposed to low noise levels. Women who scored high on both variables had a 23 mg/dL higher mean cholesterol level [95% CI 1.5 to 42.9) vs. women who scored low	⨁◯◯◯ Very low	CRITICAL
**Atrial fibrillation (assessed with previous diagnosis or study ECG)**									
1	non-randomized studies	extremely serious ^a^	not serious	not serious	not serious	none	One study (**Hahad [33]** [*n* = 14,639]) found that study participants who reported “extreme annoyance” had a higher prevalence of AF vs. participants who reported “no annoyance” (14.6 vs. 23.4%)	⨁◯◯◯ Very low	CRITICAL
**Arterial hypertension (stiffness) (assessed with carotid–femoral pulse wave velocity)**									
1	non-randomized studies	extremely serious ^a^	not serious	not serious	not serious	none	One study (**Rojek [42]** [*n* = 201]) found a positive association between noise annoyance and AH in normotensive participants and no association between noise annoyance and AH in hypertensive participants. All study participants; mean (SD) 0 (not annoyed): 9.25 m/s (1.02) 1 (moderately annoyed): 10.35 m/s (1.42) 2 (highly annoyed): 10.07 m/s (0.9) Normotensive study participants; mean (SD) 0 (not annoyed): 8.93 m/s (1.08) 1 (moderately annoyed): 10.3 m/s (1.14) 2 (highly annoyed): 9.96 m/s (0.93) Hypertensive study participants; mean (SD) 0 (not annoyed): 9.62 m/s (1.07) 1 (moderately annoyed): 9.95 m/s (1.28) 2 (highly annoyed): 10.31 m/s (1.13)	⨁◯◯◯ Very low	CRITICAL
**Cortisol (assessed with saliva and hair samples)**									
2	non-randomized studies	extremely serious ^a^	not serious	not serious	not serious	none	One study (**Wallas [24]** [*n* = 1751]) found generally positive trends in saliva cortisol levels related to noise annoyance from different sources. However, none of the trends were statistically significant following adjustment for sex, age, and sampling season, which were the only significant covariates in the models. One study (**Michaud [38]** [*n* = 1238] reported a statistically significant difference in hair cortisol levels when comparing a highly annoyed group to a group reporting low annoyance. High annoyance: geometric mean 169.79 ng/g (95% CI 124.86 to 230.89) Low annoyance: geometric mean 127.64 ng/g (95% CI 112.60 to 144.68)	⨁◯◯◯ Very low	CRITICAL
**Heart rate variability (assessed with ECG and finger plethysmograph)**									
2	non-randomized studies	extremely serious ^a^	not serious	not serious	not serious	none	Two studies (**Gallasch [32]** [*n* = 59], **Huang [35]** [*n* = 20]) found no association between noise annoyance and heart rate; results were not reported quantitatively	⨁◯◯◯ Very low	CRITICAL
**Pro-atrial natriuretic peptide (assessed with blood samples)**									
1	non-randomized studies	extremely serious ^a^	not serious	not serious	not serious	none	**Hahad [34]:** (*n* = 4826) Beta estimate for pro-ANP per point increase in annoyance Daytime annoyance: 0.014 pmol/L (95% CI 0.0057 to 0.023) Nighttime annoyance: 0.011 pmol/L (95% CI 0.0019 to 0.020) All annoyance: 0.013 pmol/L (95% CI 0.0041 to 0.021)	⨁◯◯◯ Very low	CRITICAL
**Adrenaline and noradrenaline (assessed with urine samples)**									
2	non-randomized studies	extremely serious ^a^	not serious	not serious	not serious	none	**Babisch [27]:** (*n* = 234) “Highly disturbed” vs. “less disturbed” Communication: disturbance regression coefficient 1.0 ug/g creatinine Sleep: disturbance regression coefficient 5.57 ug/g creatinine Relaxation: disturbance regression coefficient 0.59 ug/g creatinine **Arvidsson [22]:** (*n* = 50) “Quite annoyed” vs. “not especially annoyed”, mean (SD) Adrenaline: 12.5 ng/min (6.6) vs. 17.6 ng/min (7.5) Noradrenaline: 29.3 ng/min (12.1) vs. 36.3 ng/min (10.7)	⨁◯◯◯ Very low	CRITICAL
**Glomerular filtration rate (assessed with blood samples)**									
2	non-randomized studies	very serious ^c,f^	not serious	not serious	serious ^g^	none	**Dzhambov [30]:** (*n* = 217) Adjusted eGFR change: 0.20 mL/min/1.73 m^2^ (95% CI -3.97 to 4.36) per one interquartile range increase in global noise annoyance **Kannenkeril [36]:** (*n* = 113) Annoyed vs. not annoyed, mean (SD) 133 mL/min (12) vs. 138 mL/min (15)	⨁◯◯◯ Very low	CRITICAL
**Alpha-amylase (assessed with saliva samples)**									
1	non-randomized studies	extremely serious ^a^	not serious	not serious	serious ^h^	none	One study (**Gallasch [32]** [*n* = 59]) found no association between noise annoyance and salivary alpha-amylase; results were not reported quantitatively	⨁◯◯◯ Very low	CRITICAL
**Renal plasma flow (assessed with constant-infusion input clearance technique)**									
1	non-randomized studies	extremely serious ^a^	not serious	not serious	serious ^h^	none	One study (**Kannenkeril [36]** [*n* = 113]) found that noise-annoyed individuals showed lower RPF (599 ± 106 vs. 663 ± 124 mL/min), lower renal blood flow (1,068 ± 203 vs. 1,172 ± 225 mL/min), higher filtration fraction (22.7 ± 3.3 vs. 21.3 ± 3.0), higher renal vascular resistance (88.9 ± 25.6 vs. 75.8 ± 22.9 mm Hg/[mL/min]), and higher resistance of afferent arteriole (2439.5 ± 1253.4 vs. 1849.9 ± 1242.0 dyn s^−1^ cm^−5^) compared to non-noise-annoyed individuals.	⨁◯◯◯ Very low	CRITICAL

CI: confidence interval; OR: odds ratio; SD: standard deviation. Explanations: a: Rated down for risk of bias as study or studies are at serious or critical risk of bias due to lack of measurement of/control for pre-specified critical confounders and concerns with measurement of the exposure; one study (Melamed, [21]) compared high annoyance in high-noise setting vs. low annoyance in a low-noise setting; effects of annoyance cannot be separated from the actual setting variables, including noise level. b: Rated down for inconsistency because the studies vary in estimate of effect of noise annoyance on hypertension prevalence, with some reporting a positive association and others reporting no association. c: Rated down for risk of bias as Dzhambov [30] has some concerns with risk of bias due to measurement of ethnicity (Bulgarian vs. not) and lack of adjustment for sleep disturbance in the model reported. d: Alimohammadi [26] and Lercher [37] are at high risk due to a lack of measurement of/control for pre-specified critical confounders and concerns with measurement of the exposure. e: Rated down for imprecision because the 95% confidence interval of eGFR change reported by Dzhambov [30] includes both a decrease and an increase in eGFR with increasing noise annoyance. f: Rated down for risk of bias as Kannenkeril [36] has a very high risk of bias due to lack of measurement of/control for pre-specified critical confounders and concerns with measurement of the exposure. g: Rated down for imprecision as the 95% confidence interval of SBP change reported by Lercher [37] includes both a decrease and an increase in SBP with increasing noise annoyance. h: Rated down due to concerns with imprecision due to a small number of study participants and optimal information size (OIS).

## Data Availability

No new data were created or analyzed in this study.

## Supplementary Materials

The following supporting information can be downloaded at https://www.mdpi.com/article/10.3390/ijerph21070956/s1: Table S1: Literature search strategies, Table S2. List of ineligible studies.
