# Supplementary material for: A Systematic Review and Meta-Analysis of Noise Annoyance as a Determinant of Physiological Changes Linked to Disease Promotion
Source: Int J Environ Res Public Health. 2024 Jul 22;21(7):956. doi: 10.3390/ijerph21070956 (PMC11276945; doi:10.3390/ijerph21070956)
Supplement: Supplementary file 1 [file ijerph-21-00956-s001.zip › ijerph-3061654-supplementary.pdf]

Supplemental material

Table S1. Literature search strategies

PubMed

SEARCH DATE: August 28, 2023

Set	Search Strategy	Results
1	"Noise"[Mesh]	27,160
2	(noise[tiab] OR noises[tiab] OR noisy[tiab] OR infrasound*[tiab] OR "infra sound"[tiab])	184,313
3	((interrupt*[tiab] OR intrudance[tiab] OR intrude[tiab] OR intruded[tiab] OR intrusio*[tiab] OR intrusiv*[tiab] OR "low frequency"[tiab] OR undesir*[tiab] OR unwanted[tiab] OR unwelcome[tiab]) AND ("Sound"[Mesh] OR sound[tiab]))	4,586
4	(("Acoustics"[Mesh] OR "Sound"[Mesh] OR acoustic*[tiab] OR audio[tiab] OR auditory[tiab] OR sound*[tiab]) AND (densit*[tiab] OR energ*[tiab] OR expos*[tiab] OR intensit*[tiab] OR power*[tiab] OR pressure*[tiab]))	82,531
**5**	**OR/1-4**	**257,620**
6	"Irritable Mood"[Mesh]	2,036
7	(aggravat*[tiab] OR annoy*[tiab] OR exasperat*[tiab] OR irritat*[tiab] OR nuisance*[tiab] OR vexat*[tiab])	97,805
**8**	**6 OR 7**	**99,691**
**9**	**5 AND 8**	**2,199**
10	"Allostasis"[Mesh] OR "Stress, Physiological"[Mesh]	265,761
11	(allosta*[tiab] OR stress[tiab] OR stressor*[tiab])	1,009,560
12	((accumulat*[tiab] or chronic*[tiab]) AND neuroendocrine[tiab])	5,735
**13**	**OR/10-12**	**1,066,119**
14	"Cardiovascular Diseases"[Mesh]	2,720,268
15	(aneurysm*[tiab] OR arrhythmia*[tiab] OR atria*[tiab] OR "blood pressure"[tiab] OR "blood vessel"[tiab] OR "blood vessels"[tiab] OR bradycard*[tiab] OR "brugada syndrome"[tiab] OR cardia*[tiab] OR cardio*[tiab] OR cerebrovasc*[tiab] OR "commotio cordis"[tiab] OR "conduction disturbance"[tiab] OR "conduction disturbances"[tiab] OR coronary[tiab] OR fibrillat*[tiab] OR dysrhythmia*[tiab] OR heart[tiab] OR hypertensi*[tiab] OR infarct*[tiab] OR ischemi*[tiab] OR "long QT syndrome"[tiab] OR myocard*[tiab] OR NSTEMI[tiab] OR parasystole[tiab] OR "paroxysmal dyspnea"[tiab] OR "pre-excitation syndrome"[tiab] OR STEMI[tiab] OR stroke[tiab] OR tachycardia*[tiab] OR vascula*[tiab] OR vasomotor*[tiab] OR ventric*[tiab])	3,864,409
**16**	**14 OR 15**	**4,677,007**
17	("Amylases"[Mesh] OR "Biomarkers"[Mesh])	907,511

18	(amylase*[tiab] OR "biochemical marker"[tiab] OR "biochemical markers"[tiab] OR "biological marker"[tiab] OR "biological markers"[tiab] OR biomarker*[tiab] OR catecholamine[tiab] OR "clinical marker"[tiab] OR "clinical markers"[tiab] OR diastas*[tiab] OR "immune marker"[tiab] OR "immune markers"[tiab] OR "immunologic marker"[tiab] OR "immunologic markers"[tiab] OR "laboratory marker"[tiab] OR "laboratory markers"[tiab] OR "serum marker"[tiab] OR "serum markers"[tiab] OR "surrogate end point"[tiab] OR "surrogate end points"[tiab] OR "surrogate marker"[tiab] OR "surrogate markers"[tiab] OR "viral marker"[tiab] OR "viral markers"[tiab])	543,021
19	("Adiponectin"[Mesh] OR "Adrenocorticotropic Hormone"[Mesh] OR "C-Peptide"[Mesh] OR "Glucose Tolerance Test"[Mesh] OR "Glycated Hemoglobin"[Mesh] OR "Insulin"[Mesh])	313,689
20	("ACRP30 protein"[tiab] OR ACTH[tiab] OR "adipocyte complement related protein 30 kDa"[tiab] OR "adipose most abundant gene transcript 1"[tiab] OR adiponectin[tiab] OR "adrenocorticotrophic hormone"[tiab] OR "adrenocorticotropic hormone"[tiab] OR adrenocorticotropin[tiab] OR "apM-1 protein"[tiab] OR corticotrophin[tiab] OR corticotropin[tiab] OR corticotropin[tiab] OR "c-peptide"[tiab] OR "connecting peptide"[tiab] OR "fasting plasma glucose"[tiab] OR "fructated hemoglobin"[tiab] OR "fructated hemoglobins"[tiab] OR "glycated A1a-2 hemoglobin"[tiab] OR "glycated A1a-2 hemoglobins"[tiab] OR "glycated A1b hemoglobin"[tiab] OR "glycated A1b hemoglobins"[tiab] OR "glycated hemoglobin"[tiab] OR "glycated hemoglobins"[tiab] OR glycohemoglobin"[tiab] OR glycohemoglobins"[tiab] OR "glycosylated A1a-1 hemoglobin"[tiab] OR "glycosylated A1a-1 hemoglobins"[tiab] OR "glycosylated A1b hemoglobin"[tiab] OR "glycosylated A1b hemoglobins"[tiab] OR "glycosylated hemoglobin"[tiab] OR "glycosylated hemoglobins"[tiab] OR "Hb A1"[tiab] OR "Hb A1a+b"[tiab] OR "Hb A1a-1"[tiab] OR "Hb A1a-2"[tiab] OR "Hb A1b"[tiab] OR "Hb A1c"[tiab] OR "Hb A1c"[tiab] OR "HbA1"[tiab] OR HbA1c[tiab] OR "hemoglobin A(1)"[tiab] OR iletin[tiab] OR insulin[tiab] OR novolin[tiab] OR "OGTT"[tiab] OR "oral glucose tolerance"[tiab] OR "urine albumin-to-creatinine ratio"[tiab])	452,693
21	("Aldosterone"[Mesh] OR "Adrenomedullin"[Mesh] OR "Atrial Natriuretic Factor"[Mesh] OR "Brain-Derived Neurotrophic Factor"[Mesh] OR "C-Reactive Protein"[Mesh] OR "Endothelin-1"[Mesh] OR "Epinephrine"[Mesh] OR "Fibrinogen"[Mesh] OR "Galectin 3"[Mesh] OR "Homocysteine"[Mesh] OR "Hydrocortisone"[Mesh] OR "Hypertension"[Mesh] OR "Lipids"[Mesh] OR "Natriuretic Peptide, Brain"[Mesh] OR "Norepinephrine"[Mesh] OR "Peroxidase"[Mesh] OR "Troponin"[Mesh] OR "Tumor Necrosis Factor-alpha"[Mesh] OR "fibrin fragment D"[Supplementary Concept] OR "oxidized low density lipoprotein"[Supplementary Concept])	865,152

22	("18-Oxocorticosterone"[tiab] OR "4-α-D-glucan glucanohydrolase"[tiab] OR "α-Amylase"[tiab] OR "a-amylase"[tiab] OR ACTH[tiab] OR adrenaline[tiab] OR "adrenocorticotropic hormone"[tiab] OR aldocorten[tiab] OR aldocortene[tiab] OR aldocortin[tiab] OR aldosterone[tiab] OR aldosteronum[tiab] OR "alpha-amylase"[tiab] OR "alpha ANP"[tiab] OR adrenomedullin[tiab] OR arterenol[tiab] OR "atrial natriuretic factor"[tiab] OR "atrial natriuretic peptide"[tiab] OR "atrial natriuretic peptides"[tiab] OR "atrial pronatriodilatin"[tiab] OR atriopeptigen[tiab] OR atriopeptins[tiab] OR BDNF[tiab] OR "beta ANP"[tiab] OR "big endothelin"[tiab] OR "BNP"[tiab] OR "brain-derived neurotrophic factor"[tiab] OR "brain natriuretic peptide"[tiab] OR cachectin[tiab] OR "carbohydrate binding protein 35"[tiab] OR "cardiodilatin precursor"[tiab] OR "cardionatrin I"[tiab] OR "cardionatrin IV"[tiab] OR "cathechol amine*"[tiab] OR cathecholamin*[tiab] OR "CBP30"[tiab] OR "CBP-30"[tiab] OR "CBP35"[tiab] OR "CBP-35"[tiab] OR ceroid[tiab] OR "coagulation factor I"[tiab] OR cortef[tiab] OR corticotropin[tiab] OR cortifair[tiab] OR cortifair[tiab] OR cortisol[tiab] OR cortril[tiab] OR cortrophin[tiab] OR "creactive protein*"[tiab] OR "c-reactive protein*"[tiab] OR CRP[tiab] OR curiculin[tiab] OR "d dimer"[tiab] OR electrocortin[tiab] OR "electronegative low-density lipoprotein"[tiab] OR elektrocortin[tiab] OR "endothelin-1"[tiab] OR "endothelin type 1"[tiab] OR epicortisol[tiab] OR epifrin[tiab] OR epinephrine[tiab] OR epitrate[tiab] OR "epsilon binding protein"[tiab] OR "ET-1"[tiab] OR "ET1"[tiab] OR fats[tiab] OR "fatty acid"[tiab] OR "fatty acids"[tiab] OR "fatty alcohol"[tiab] OR "fatty alcohols"[tiab] OR "fibrin fragment D"[tiab] OR fibrinogen[tiab] OR "galectin 3"[tiab] OR "gamma ANP"[tiab] OR glyceride*[tiab] OR glycolipid*[tiab] OR "heart rate variability"[tiab] OR "HL29"[tiab] OR "HL-29"[tiab] OR homocysteine[tiab] OR "HRV"[tiab] OR hsCRP[tiab] OR hydrocortisone[tiab] OR hypertension[tiab] OR "IgE Binding Protein"[tiab] OR "IgEBP"[tiab] OR "L29 Lectin"[tiab] OR "L-29 Lectin"[tiab] OR "L30 Lectin"[tiab] OR "L-30 Lectin"[tiab] OR "L31"[tiab] OR "L-31"[tiab] OR "L34"[tiab] OR "L-34"[tiab] OR levarterenol[tiab] OR levonor[tiab] OR levonorepinephrine[tiab] OR levophed[tiab] OR "LGALS3"[tiab] OR lipid[tiab] OR lipids[tiab] OR lipofuscin[tiab] OR lipopeptide*[tiab] OR lipopolysaccharide*[tiab] OR lipoprotein*[tiab] OR lyophrin[tiab] OR "mac 2 antigen"[tiab] OR "macrophage 2 antigen"[tiab] OR "medihaler-epi"[tiab] OR "minimally modified oxidized-LDL"[tiab] OR "MM-LDL"[tiab] OR myeloperoxidase[tiab] OR noradrenaline[tiab] OR "norepinephrine"[tiab] OR "oxidized low density lipoprotein"[tiab] OR "oxidized LDL"[tiab] OR "ox-LDL"[tiab] OR OxLDL[tiab] OR peroxidase[tiab] OR "prepro ANP"[tiab] OR "prepro CDD ANF"[tiab] OR "pro ANF"[tiab] OR "proANF"[tiab] OR "proatrial natriuretic factor"[tiab] OR pronatriodilatin[tiab] OR pyrocatechinamine*[tiab] OR pyrocatecholamine[tiab] OR "Reichstein X"[tiab] OR sCortisol[tiab] OR "sICAM-1"[tiab] OR "soluble intercellular adhesion molecule-1"[tiab] OR "TNF-α"[tiab] OR "TNF-a"[tiab] OR TNFα[tiab] OR TNFa[tiab] OR "TNF-alpha"[tiab] OR TNFalpha[tiab] OR troponin[tiab] OR "tumor necrosis factor-alpha"[tiab])	2,261,111

23	("8-Hydroxy-2'-Deoxyguanosine"[Mesh] OR "Advanced Oxidation Protein Products"[Mesh] OR "Catalase"[Mesh] OR "Glutathione Peroxidase"[Mesh] OR "Malondialdehyde"[Mesh] OR "Nitric Oxide"[Mesh] OR "Reactive Oxygen Species"[Mesh] OR "Superoxide Dismutase"[Mesh])	391,908
24	("8-hydroxy-2'-deoxyguanosine"[tiab] OR "8OHdG"[tiab] OR "8-OHdG"[tiab] OR "active oxygen"[tiab] OR "advanced oxidation protein products"[tiab] OR catalase[tiab] OR "endogenous nitrate vasodilator"[tiab] OR "endothelium-derived nitric oxide"[tiab] OR erythrocuprein[tiab] OR "glutathione lipoperoxidase"[tiab] OR "glutathione peroxidase"[tiab] OR hemocuprein[tiab] OR malonaldehyde[tiab] OR malondialdehyde[tiab] OR malonylaldehyde[tiab] OR malonyldialdehyde[tiab] OR "Mn-SOD"[tiab] OR "mononitrogen monoxide"[tiab] OR "nitric oxide"[tiab] OR "nitrogen monoxide"[tiab] OR "oxygen radical"[tiab] OR "oxygen radicals"[tiab] OR "pro-oxidant"[tiab] OR "pro-oxidants"[tiab] OR propanedial[tiab] OR "reactive oxygen intermediates"[tiab] OR "reactive oxygen species"[tiab] OR "selenoglutathione peroxidase"[tiab] OR "superoxide dismutase"[tiab] OR "total antioxidant capacity"[tiab])	464,413
**25**	**OR/17-24**	**4,186,628**
26	"Cytokines"[Mesh]	792,020
27	(adipokines[tiab] OR chemokine*[tiab] OR cytokine*[tiab] OR interferon*[tiab] OR interleukin*[tiab] OR IL1[tiab] OR IL2[tiab] OR IL3[tiab] OR IL4[tiab] OR IL5[tiab] OR IL6[tiab] OR IL7[tiab] OR IL8[tiab] OR IL9[tiab] OR "IL-1"[tiab] OR "IL-2"[tiab] OR"IL-3"[tiab] OR"IL-4"[tiab] OR"IL-5"[tiab] OR"IL-6"[tiab] OR"IL-7"[tiab] OR"IL-8"[tiab] OR"IL-9"[tiab] OR lymphokine*[tiab] OR mesenchymal growth factor*[tiab] OR MGF[tiab] OR monokine*[tiab] OR TNF[tiab] OR tumor necrosis factor* tumour necrosis factor*)	968,185
**28**	**26 OR 27**	**1,193,167**
29	"Biological Monitoring"[Mesh]	1,148
30	("biological monitor*"[tiab] OR "biologic monitor*"[tiab] OR biomonitor*[tiab] OR "bio-monitor*"[tiab])	14,241
31	("Hair Analysis"[Mesh] OR "Hematologic Tests"[Mesh] OR "Urinalysis"[Mesh] OR (("Blood"[Mesh] OR "Feces"[Mesh] OR "Hair"[Mesh] OR "Milk, Human"[Mesh] OR "Saliva"[Mesh] OR "Sputum"[Mesh] OR "Urine"[Mesh]) AND ("analysis" [Subheading] OR analy*[tiab] OR assay*[tiab] OR concentration*[tiab] OR level*[tiab] OR sampl*[tiab] OR test*[tiab])))	1,138,990
32	(("breast milk"[tiab]OR blood[tiab] OR faecal[tiab] OR faeces[tiab] OR fecal[tiab] OR feces[tiab] OR hair[tiab] OR haematolog*[tiab] OR hematolog*[tiab] OR "human milk"[tiab] OR plasma[tiab] OR saliv*[tiab] OR sera[tiab] OR serolog*[tiab] OR serum[tiab] OR sputum*[tiab] OR stool[tiab] OR urina*[tiab] OR urine*[tiab]) AND (analy*[tiab] OR concentration*[tiab] OR level*[tiab] OR sampl*[tiab] OR test*[tiab]))	3,065,527
**33**	**OR/29-32**	**3,729,386**
**34**	**13 OR 16 OR 25 OR 28 OR 33**	**10,673,601**

**35**	**9 AND 34**	**599**
36	(english[Filter])	
**37**	**35 AND 36**	**535**
38	(("Clinical Study"[Publication Type] OR "Comparative Study"[Publication Type] OR "Multicenter Study"[Publication Type] OR compara*[tiab] OR compare*[tiab] OR compari*[tiab] OR placebo[tiab] OR random*[tiab] OR trial*[ti] OR (case[tiab] AND (control[tiab] OR report*[tiab] OR series[tiab] OR studies[tiab] OR study[tiab])) OR ((clinical[tiab] OR cohort[tiab] OR crossover[tiab] OR "cross-over"[tiab] OR "cross-sectional"[tiab] OR multicenter[tiab]) AND (studies[tiab] OR study[tiab] OR trial*[tiab]))) OR ("Meta-Analysis"[Publication Type] OR "Review"[Publication Type] OR "Systematic Review"[Publication Type] OR metaanalys*[ti] OR meta-analys*[ti] OR "meta analys*"[ti] OR review[ti] OR "systematic review"[ti] OR ((meta[ti] OR systematic*[ti]) AND (overview*[tiab] OR review*[tiab] OR synthesis*[tiab]))))	
**39**	**37 AND 38**	**357**

	**Cochrane**	
	**SEARCH DATE: August 28, 2023**	
**Set #**	**Search Strategy**	**Results**
1	MeSH descriptor: [Noise] explode all trees	676
2	((noise OR noises OR noisy OR infrasound* OR "infra sound"):ti,ab)	5,066
3	(((interrupt* OR intrudance OR intrude OR intruded OR intrusio* OR intrusiv* OR "low frequency" OR undesir* OR unwanted OR unwelcome):ti,ab) AND (MeSH descriptor: [Sound] explode all trees OR sound:ti,ab))	20,914
4	((MeSH descriptor: [Acoustics] explode all trees OR MeSH descriptor: [Sound] explode all trees OR ((acoustic* OR audio OR auditory OR sound*):ti,ab)) AND ((densit* OR energ* OR expos* OR intensit* OR power* OR pressure*):ti,ab))	216
**5**	**OR/1-4**	**5,307**
6	MeSH descriptor: [Irritable Mood] explode all trees	182
7	((aggravat* OR annoy* OR exasperat* OR irritat* OR nuisance* OR vexat*):ti,ab)	10,096
**8**	**6 OR 7**	**10,271**
**9**	**5 AND 8**	**93**
10	MeSH descriptor: [Allostasis] explode all trees OR MeSH descriptor: [Stress, Physiological] explode all trees	6,366
11	((allosta* OR stress OR stressor*):ti,ab)	65,896
12	(((accumulat* or chronic*) AND neuroendocrine):ti,ab)	274
**13**	**OR/10-12**	**67,051**
14	MeSH descriptor: [Cardiovascular Diseases] explode all trees	151,385

15	((aneurysm* OR arrhythmia* OR atria* OR "blood pressure" OR "blood vessel" OR "blood vessels" OR bradycard* OR "brugada syndrome" OR cardia* OR cardio* OR cerebrovasc* OR "commotio cordis" OR "conduction disturbance" OR "conduction disturbances" OR coronary OR fibrillat* OR dysrhythmia* OR heart OR hypertensi* OR infarct* OR ischemi* OR "long QT syndrome" OR myocard* OR NSTEMI OR parasystole OR "paroxysmal dyspnea" OR "pre-excitation syndrome" OR STEMI OR stroke OR tachycardia* OR vascula* OR vasomotor* OR ventric*):ti,ab)	406,415
**16**	**14 OR 15**	**435,038**
17	MeSH descriptor: [Amylases] explode all trees OR MeSH descriptor: [Biomarkers] explode all trees	27,359
18	((amylase* OR "biochemical marker" OR "biochemical markers" OR "biological marker" OR "biological markers" OR biomarker* OR catecholamine OR "clinical marker" OR "clinical markers" OR diastas* OR "immune marker" OR "immune markers" OR "immunologic marker" OR "immunologic markers" OR "laboratory marker" OR "laboratory markers" OR "serum marker" OR "serum markers" OR "surrogate end point" OR "surrogate end points" OR "surrogate marker" OR "surrogate markers" OR "viral marker" OR "viral markers"):ti,ab)	48,750
19	(MeSH descriptor: [Adiponectin] explode all trees OR MeSH descriptor: [Adrenocorticotropic Hormone] explode all trees OR MeSH descriptor: [C-Peptide] explode all trees OR MeSH descriptor: [Glucose Tolerance Test] explode all trees OR MeSH descriptor: [Glycated Hemoglobin] explode all trees OR MeSH descriptor: [Insulin] explode all trees)	24,521
20	(("ACRP30 protein" OR ACTH OR "adipocyte complement related protein 30 kDa" OR "adipose most abundant gene transcript 1" OR adiponectin OR "adrenocorticotrophic hormone" OR "adrenocorticotropic hormone" OR adrenocorticotropin OR "apM-1 protein" OR corticotrophin OR corticotropin OR corticotropin OR "c-peptide" OR "connecting peptide" OR "fasting plasma glucose" OR "fructated hemoglobin" OR "fructated hemoglobins" OR "glycated A1a-2 hemoglobin" OR "glycated A1a-2 hemoglobins" OR "glycated A1b hemoglobin" OR "glycated A1b hemoglobins" OR "glycated hemoglobin" OR "glycated hemoglobins" OR glycohemoglobin OR glycohemoglobins OR "glycosylated A1a-1 hemoglobin" OR "glycosylated A1a-1 hemoglobins" OR "glycosylated A1b hemoglobin" OR "glycosylated A1b hemoglobins" OR "glycosylated hemoglobin" OR "glycosylated hemoglobins" OR "Hb A1" OR "Hb A1a+b" OR "Hb A1a-1" OR "Hb A1a-2" OR "Hb A1b" OR "Hb A1c" OR "Hb A1c" OR "HbA1" OR HbA1c OR "hemoglobin A(1)" OR iletin OR insulin OR novolin OR "OGTT" OR "oral glucose tolerance" OR "urine albumin-to-creatinine ratio"):ti,ab)	76,548
21	(MeSH descriptor: [Aldosterone] explode all trees OR MeSH descriptor: [Adrenomedullin] explode all trees OR MeSH descriptor: [Atrial Natriuretic Factor] explode all trees OR MeSH descriptor: [Brain-Derived Neurotrophic Factor] explode all trees OR MeSH descriptor: [C-Reactive Protein] explode all	105,339

	trees OR MeSH descriptor: [Endothelin-1] explode all trees OR MeSH descriptor: [Epinephrine] explode all trees OR MeSH descriptor: [Fibrinogen] explode all trees OR MeSH descriptor: [Galectin 3] explode all trees OR MeSH descriptor: [Homocysteine] explode all trees OR MeSH descriptor: [Hydrocortisone] explode all trees OR MeSH descriptor: [Hypertension] explode all trees OR MeSH descriptor: [Lipids] explode all trees OR MeSH descriptor: [Natriuretic Peptide, Brain] explode all trees OR MeSH descriptor: [Norepinephrine] explode all trees OR MeSH descriptor: [Peroxidase] explode all trees OR MeSH descriptor: [Troponin] explode all trees OR MeSH descriptor: [Tumor Necrosis Factor-alpha] explode all trees)	
22	(("18-Oxocorticosterone" OR "4-α-D-glucan glucanohydrolase" OR "α-Amylase" OR "a-amylase" OR ACTH OR adrenaline OR "adrenocorticotropic hormone" OR aldocorten OR aldocortene OR aldocortin OR aldosterone OR aldosteronum OR "alpha-amylase" OR "alpha ANP" OR adrenomedullin OR arterenol OR "atrial natriuretic factor" OR "atrial natriuretic peptide" OR "atrial natriuretic peptides" OR "atrial pronatriodilatin" OR atriopeptigen OR atriopeptins OR BDNF OR "beta ANP" OR "big endothelin" OR "BNP" OR "brain-derived neurotrophic factor" OR "brain natriuretic peptide" OR cachectin OR "carbohydrate binding protein 35" OR "cardiodilatin precursor" OR "cardionatrin I" OR "cardionatrin IV" OR "cathechol amine" OR "cathechol amines" OR cathecholamin* OR "CBP30" OR "CBP-30" OR "CBP35" OR "CBP-35" OR ceroid OR "coagulation factor I" OR cortef OR corticotropin OR cortifair OR cortifair OR cortisol OR cortril OR cortrophin OR "creactive protein" OR "c-reactive protein" OR "creactive proteins" OR "c-reactive proteins" OR CRP OR curiculin OR "d dimer" OR electrocortin OR "electronegative low-density lipoprotein" OR elektrocortin OR "endothelin-1" OR "endothelin type 1" OR epicortisol OR epifrin OR epinephrine OR epitrate OR "epsilon binding protein" OR "ET-1" OR "ET1" OR fats OR "fatty acid" OR "fatty acids" OR "fatty alcohol" OR "fatty alcohols" OR "fibrin fragment D" OR fibrinogen OR "galectin 3" OR "gamma ANP" OR glyceride* OR glycolipid* OR "heart rate variability" OR "HL29" OR "HL-29" OR homocysteine OR "HRV" OR hsCRP OR hydrocortisone OR hypertension OR "IgE Binding Protein" OR "IgEBP" OR "L29 Lectin" OR "L-29 Lectin" OR "L30 Lectin" OR "L-30 Lectin" OR "L31" OR "L-31" OR "L34" OR "L-34" OR levarterenol OR levonor OR levonorepinephrine OR levophed OR "LGALS3" OR lipid OR lipids OR lipofuscin OR lipopeptide* OR lipopolysaccharide* OR lipoprotein* OR lyophrin OR "mac 2 antigen" OR "macrophage 2 antigen" OR "medihaler-epi" OR "minimally modified oxidized-LDL" OR "MM-LDL" OR myeloperoxidase OR noradrenaline OR "norepinephrine" OR "oxidized low density lipoprotein" OR "oxidized LDL" OR "ox-LDL" OR OxLDL OR peroxidase OR "prepro ANP" OR "prepro CDD ANF" OR "pro ANF" OR "proANF" OR "proatrial natriuretic factor" OR pronatriodilatin OR pyrocatechinamine* OR pyrocatecholamine OR "Reichstein X" OR sCortisol OR "sICAM-1" OR "soluble intercellular adhesion	209,004

	molecule-1" OR "TNF-α" OR "TNF-a" OR TNFα OR TNFa OR "TNF-alpha" OR TNFalpha OR troponin OR "tumor necrosis factor-alpha"):ti,ab)	
23	(MeSH descriptor: [8-Hydroxy-2'-Deoxyguanosine] explode all trees OR MeSH descriptor: [Advanced Oxidation Protein Products] explode all trees OR MeSH descriptor: [Catalase] explode all trees OR MeSH descriptor: [Glutathione Peroxidase] explode all trees OR MeSH descriptor: [Malondialdehyde] explode all trees OR MeSH descriptor: [Nitric Oxide] explode all trees OR MeSH descriptor: [Reactive Oxygen Species] explode all trees OR MeSH descriptor: [Superoxide Dismutase] explode all trees)	6,545
24	(("8-hydroxy-2'-deoxyguanosine" OR "8OHdG" OR "8-OHdG" OR "active oxygen" OR "advanced oxidation protein products" OR catalase OR "endogenous nitrate vasodilator" OR "endothelium-derived nitric oxide" OR erythrocuprein OR "glutathione lipoperoxidase" OR "glutathione peroxidase" OR hemocuprein OR malonaldehyde OR malondialdehyde OR malonylaldehyde OR malonyldialdehyde OR "Mn-SOD" OR "mononitrogen monoxide" OR "nitric oxide" OR "nitrogen monoxide" OR "oxygen radical" OR "oxygen radicals" OR "pro-oxidant" OR "pro-oxidants" OR propanedial OR "reactive oxygen intermediates" OR "reactive oxygen species" OR "selenoglutathione peroxidase" OR "superoxide dismutase" OR "total antioxidant capacity"):ti,ab)	16,434
**25**	**OR/17-24**	**343,862**
26	MeSH descriptor: [Cytokines] explode all trees	24,323
27	((adipokines OR chemokine* OR cytokine* OR interferon* OR interleukin* OR IL1 OR IL2 OR IL3 OR IL4 OR IL5 OR IL6 OR IL7 OR IL8 OR IL9 OR "IL-1" OR "IL-2" OR "IL-3" OR "IL-4" OR "IL-5" OR "IL-6" OR "IL-7" OR "IL-8" OR "IL-9" OR lymphokine* OR mesenchymal growth factor* OR MGF OR monokine* OR TNF OR "tumor necrosis factor" OR "tumor necrosis factors" OR "tumour necrosis factor" OR "tumour necrosis factors"):ti,ab)	68,309
**28**	**26 OR 27**	**75,905**
29	MeSH descriptor: [Biological Monitoring] explode all trees	17
30	(("biological monitor" OR "biological monitors" OR "biological monitoring" OR "biologic monitor" OR "biologic monitors" OR "biologic monitoring" OR biomonitor* OR "bio-monitor" OR "bio-monitors" OR "bio-monitoring"):ti,ab)	139
31	(MeSH descriptor: [Hair Analysis] explode all trees OR MeSH descriptor: [Hematologic Tests] explode all trees OR MeSH descriptor: [Urinalysis] explode all trees OR ((MeSH descriptor: [Blood] explode all trees OR MeSH descriptor: [Feces] explode all trees OR MeSH descriptor: [Hair] explode all trees OR MeSH descriptor: [Milk, Human] explode all trees OR MeSH descriptor: [Saliva] explode all trees OR MeSH descriptor: [Sputum] explode all trees OR MeSH descriptor: [Urine] explode all trees) AND (Any MeSH descriptor in all MeSH products and with qualifier(s): [analysis - AN] OR ((analy* OR assay* OR concentration* OR level* OR sampl* OR test*):ti,ab))))	41,196

32	((("breast milk" OR blood OR faecal OR faeces OR fecal OR feces OR hair OR haematolog* OR hematolog* OR "human milk" OR plasma OR saliv* OR sera OR serolog* OR serum OR sputum* OR stool OR urina* OR urine*):ti,ab) AND (Any MeSH descriptor in all MeSH products and with qualifier(s): [analysis - AN] OR ((analy* OR concentration* OR level* OR sampl* OR test*):ti,ab)))	20,100
**33**	**OR/29-32**	**56,237**
**34**	**13 OR 16 OR 25 OR 28 OR 33**	**712,750**
**35**	**9 AND 34**	**30**

	**Embase**	
	**SEARCH DATE: August 28, 2023**	
**Set #**	**Search Strategy**	**Results**
1	noise'/exp	147,334
2	((noise OR noises OR noisy OR infrasound* OR "infra sound"):ti,ab)	199,689
3	(((interrupt* OR intrudance OR intrude OR intruded OR intrusio* OR intrusiv* OR "low frequency" OR undesir* OR unwanted OR unwelcome):ti,ab) AND ('sound'/exp OR sound:ti,ab))	7,110
4	(('acoustics'/exp OR 'sound'/exp OR ((acoustic* OR audio OR auditory OR sound*):ti,ab)) AND ((densit* OR energ* OR expos* OR intensit* OR power* OR pressure*):ti,ab))	130,478
**5**	**OR/1-4**	**355,927**
6	irritability'/exp	30,336
7	((aggravat* OR annoy* OR exasperat* OR irritat* OR nuisance* OR vexat*):ti,ab)	137,580
**8**	**6 OR 7**	**167,356**
**9**	**5 AND 8**	**3,639**
10	allostasis'/exp OR 'physiological stress'/exp	498,857
11	((allosta* OR stress OR stressor*):ti,ab)	1,222,459
12	(((accumulat* or chronic*) AND neuroendocrine):ti,ab)	8,275
**13**	**OR/10-12**	**1,407,861**
14	cardiovascular disease'/exp	5,450,701
15	((aneurysm* OR arrhythmia* OR atria* OR "blood pressure" OR "blood vessel" OR "blood vessels" OR bradycard* OR "brugada syndrome" OR cardia* OR cardio* OR cerebrovasc* OR "commotio cordis" OR "conduction disturbance" OR "conduction disturbances" OR coronary OR fibrillat* OR dysrhythmia* OR heart OR hypertensi* OR infarct* OR ischemi* OR "long QT syndrome" OR myocard* OR NSTEMI OR parasystole OR "paroxysmal dyspnea" OR "pre-excitation syndrome" OR STEMI OR stroke OR tachycardia* OR vascula* OR vasomotor* OR ventric*):ti,ab)	5,362,631

**16**	**14 OR 15**	**7,252,535**
17	amylase'/exp OR 'biological marker'/exp	481,962
18	((amylase* OR "biochemical marker" OR "biochemical markers" OR "biological marker" OR "biological markers" OR biomarker* OR catecholamine OR "clinical marker" OR "clinical markers" OR diastas* OR "immune marker" OR "immune markers" OR "immunologic marker" OR "immunologic markers" OR "laboratory marker" OR "laboratory markers" OR "serum marker" OR "serum markers" OR "surrogate end point" OR "surrogate end points" OR "surrogate marker" OR "surrogate markers" OR "viral marker" OR "viral markers"):ti,ab)	767,033
19	('adiponectin'/exp OR 'corticotropin'/exp OR 'C peptide'/exp OR 'glucose tolerance test'/exp OR 'glycated hemoglobin'/exp OR 'insulin'/exp)	667,601
20	(("ACRP30 protein" OR ACTH OR "adipocyte complement related protein 30 kDa" OR "adipose most abundant gene transcript 1" OR adiponectin OR "adrenocorticotrophic hormone" OR "adrenocorticotropic hormone" OR adrenocorticotropin OR "apM-1 protein" OR corticotrophin OR corticotropin OR corticotropin OR "c-peptide" OR "connecting peptide" OR "fasting plasma glucose" OR "fructated hemoglobin" OR "fructated hemoglobins" OR "glycated A1a-2 hemoglobin" OR "glycated A1a-2 hemoglobins" OR "glycated A1b hemoglobin" OR "glycated A1b hemoglobins" OR "glycated hemoglobin" OR "glycated hemoglobins" OR glycohemoglobin OR glycohemoglobins OR "glycosylated A1a-1 hemoglobin" OR "glycosylated A1a-1 hemoglobins" OR "glycosylated A1b hemoglobin" OR "glycosylated A1b hemoglobins" OR "glycosylated hemoglobin" OR "glycosylated hemoglobins" OR "Hb A1" OR "Hb A1a+b" OR "Hb A1a-1" OR "Hb A1a-2" OR "Hb A1b" OR "Hb A1c" OR "Hb A1c" OR "HbA1" OR HbA1c OR "hemoglobin A(1)" OR iletin OR insulin OR novolin OR "OGTT" OR "oral glucose tolerance" OR "urine albumin-to-creatinine ratio"):ti,ab)	728,002
21	('adrenomedullin'/exp OR 'aldosterone'/exp OR 'atrial natriuretic factor'/exp OR 'brain derived neurotrophic factor'/exp OR 'brain natriuretic peptide'/exp OR 'C reactive protein'/exp OR 'endothelin 1'/exp OR 'epinephrine'/exp OR 'fibrin degradation product'/exp OR 'fibrinogen'/exp OR 'galectin 3'/exp OR 'homocysteine'/exp OR 'hydrocortisone'/exp OR 'hypertension'/exp OR 'lipid'/exp OR 'noradrenalin'/exp OR 'oxidized low density lipoprotein'/exp OR 'peroxidase'/exp OR 'troponin'/exp OR 'tumor necrosis factor'/exp)	4,030,504
22	(("18-Oxocorticosterone" OR "4-α-D-glucan glucanohydrolase" OR "α-Amylase" OR "a-amylase" OR ACTH OR adrenaline OR "adrenocorticotropic hormone" OR aldocorten OR aldocortene OR aldocortin OR aldosterone OR aldosteronum OR "alpha-amylase" OR "alpha ANP" OR adrenomedullin OR arterenol OR "atrial natriuretic factor" OR "atrial natriuretic peptide" OR "atrial natriuretic peptides" OR "atrial pronatriodilatin" OR atriopeptigen OR atriopeptins OR BDNF OR "beta ANP" OR "big endothelin" OR "BNP" OR "brain-derived neurotrophic factor" OR "brain natriuretic peptide" OR cachectin OR "carbohydrate binding protein 35" OR "cardiodilatin precursor"	2,969,277

	OR "cardionatrin I" OR "cardionatrin IV" OR "cathechol amine*" OR cathecholamin* OR "CBP30" OR "CBP-30" OR "CBP35" OR "CBP-35" OR ceroid OR "coagulation factor I" OR cortef OR corticotropin OR cortifair OR cortifair OR cortisol OR cortril OR cortrophin OR "creactive protein*" OR "c-reactive protein*" OR CRP OR curiculin OR "d dimer" OR electrocortin OR "electronegative low-density lipoprotein" OR elektrocortin OR "endothelin-1" OR "endothelin type 1" OR epicortisol OR epifrin OR epinephrine OR epitrate OR "epsilon binding protein" OR "ET-1" OR "ET1" OR fats OR "fatty acid" OR "fatty acids" OR "fatty alcohol" OR "fatty alcohols" OR "fibrin fragment D" OR fibrinogen OR "galectin 3" OR "gamma ANP" OR glyceride* OR glycolipid* OR "heart rate variability" OR "HL29" OR "HL-29" OR homocysteine OR "HRV" OR hsCRP OR hydrocortisone OR hypertension OR "IgE Binding Protein" OR "IgEBP" OR "L29 Lectin" OR "L-29 Lectin" OR "L30 Lectin" OR "L-30 Lectin" OR "L31" OR "L-31" OR "L34" OR "L-34" OR levarterenol OR levonor OR levonorepinephrine OR levophed OR "LGALS3" OR lipid OR lipids OR lipofuscin OR lipopeptide* OR lipopolysaccharide* OR lipoprotein* OR lyophrin OR "mac 2 antigen" OR "macrophage 2 antigen" OR "medihaler-epi" OR "minimally modified oxidized-LDL" OR "MM-LDL" OR myeloperoxidase OR noradrenaline OR "norepinephrine" OR "oxidized low density lipoprotein" OR "oxidized LDL" OR "ox-LDL" OR OxLDL OR peroxidase OR "prepro ANP" OR "prepro CDD ANF" OR "pro ANF" OR "proANF" OR "proatrial natriuretic factor" OR pronatriodilatin OR pyrocatechinamine* OR pyrocatecholamine OR "Reichstein X" OR sCortisol OR "sICAM-1" OR "soluble intercellular adhesion molecule-1" OR "TNF-α" OR "TNF-a" OR TNFα OR TNFa OR "TNF-alpha" OR TNFalpha OR troponin OR "tumor necrosis factor-alpha"):ti,ab)	
23	('8 hydroxydeoxyguanosine'/exp OR 'advanced oxidation protein product'/exp OR 'catalase'/exp OR 'glutathione peroxidase'/exp OR 'malonaldehyde'/exp OR 'nitric oxide'/exp OR 'reactive oxygen metabolite'/exp OR 'superoxide dismutase'/exp)	573,041
24	(("8-hydroxy-2-deoxyguanosine" OR "8OHdG" OR "8-OHdG" OR "active oxygen" OR "advanced oxidation protein products" OR catalase OR "endogenous nitrate vasodilator" OR "endothelium-derived nitric oxide" OR erythrocuprein OR "glutathione lipoperoxidase" OR "glutathione peroxidase" OR hemocuprein OR malonaldehyde OR malondialdehyde OR malonylaldehyde OR malonyldialdehyde OR "Mn-SOD" OR "mononitrogen monoxide" OR "nitric oxide" OR "nitrogen monoxide" OR "oxygen radical" OR "oxygen radicals" OR "pro-oxidant" OR "pro-oxidants" OR propanedial OR "reactive oxygen intermediates" OR "reactive oxygen species" OR "selenoglutathione peroxidase" OR "superoxide dismutase" OR "total antioxidant capacity"):ti,ab)	548,936
**25**	**OR/17-24**	**6,452,575**
26	cytokine'/exp	1,944,754

27	((adipokines OR chemokine* OR cytokine* OR interferon* OR interleukin* OR IL1 OR IL2 OR IL3 OR IL4 OR IL5 OR IL6 OR IL7 OR IL8 OR IL9 OR "IL-1" OR "IL-2" OR "IL-3" OR "IL-4" OR "IL-5" OR "IL-6" OR "IL-7" OR "IL-8" OR "IL-9" OR lymphokine* OR mesenchymal growth factor* OR MGF OR monokine* OR TNF OR "tumor necrosis factor" OR "tumor necrosis factors" OR "tumour necrosis factor" OR "tumour necrosis factors"):ti,ab)	488,842
**28**	**26 OR 27**	**2,029,048**
29	biological monitoring'/exp	20,164
30	(("biological monitor" OR "biological monitors" OR "biological monitoring" OR "biologic monitor" OR "biologic monitors" OR "biologic monitoring" OR biomonitor* OR "bio-monitor" OR "bio-monitors" OR "bio-monitoring"):ti,ab)	15,358
31	('blood examination'/exp OR 'hair analysis'/exp OR 'urinalysis'/exp OR (('blood'/exp OR 'breast milk'/exp OR 'feces'/exp OR 'hair'/exp OR 'saliva'/exp OR 'sputum'/exp OR 'urine'/exp) AND ('analysis'/exp OR ((analy* OR assay* OR concentration* OR level* OR sampl* OR test*):ti,ab))))	2,406,160
32	((("breast milk" OR blood OR faecal OR faeces OR fecal OR feces OR hair OR haematolog* OR hematolog* OR "human milk" OR plasma OR saliv* OR sera OR serolog* OR serum OR sputum* OR stool OR urina* OR urine*):ti,ab) AND ('analysis'/exp OR ((analy* OR concentration* OR level* OR sampl* OR test*):ti,ab)))	4,752,115
**33**	**OR/29-32**	**5,635,353**
**34**	**13 OR 16 OR 25 OR 28 OR 33**	**15,566,061**
**35**	**9 AND 34**	**1,218**
36	[english]/lim	
**37**	**35 AND 36**	**1,083**
38	(('clinical study'/exp OR 'comparative study'/exp OR ((compara* OR compare* OR compari* OR placebo OR random*):ti,ab) OR trial*:ti OR (case:ti,ab AND ((control OR report* OR series OR studies OR study):ti,ab)) OR (((clinical OR cohort OR crossover OR "cross-over" OR "cross-sectional" OR multicenter):ti,ab) AND ((studies OR study OR trial*):ti,ab))) OR ('meta analysis'/exp OR 'review'/exp OR ((metaanalys* OR meta-analys* OR "meta analys*" OR review OR "systematic review"):ti) OR (((meta OR systematic*) AND (overview* OR review* OR synthesis*)):ti)))	
**39**	**37 AND 38**	**805**

	**CINAHL**	
	**SEARCH DATE: August 28, 2023**	
**Set #**	**Search Strategy**	**Results**
1	MH "Noise"	8,588

2	((AB (noise OR noises OR noisy OR infrasound* OR "infra sound")) OR (TI (noise OR noises OR noisy OR infrasound* OR "infra sound")))	20,429
3	(((AB (interrupt* OR intrudance OR intrude OR intruded OR intrusio* OR intrusiv* OR "low frequency" OR undesir* OR unwanted OR unwelcome)) OR (TI (interrupt* OR intrudance OR intrude OR intruded OR intrusio* OR intrusiv* OR "low frequency" OR undesir* OR unwanted OR unwelcome))) AND (MH "Sound+" OR sound:ti,ab))	349
4	((MH "Acoustics+" OR MH "Sound+" OR (AB (acoustic* OR audio OR auditory OR sound*):ti,ab) OR (TI (acoustic* OR audio OR auditory OR sound*))) AND ((AB (densit* OR energ* OR expos* OR intensit* OR power* OR pressure*)) OR (TI (densit* OR energ* OR expos* OR intensit* OR power* OR pressure*))))	5,387
**5**	**OR/1-4**	**25,734**
6	MH "Emotional Lability"	42
7	((AB (aggravat* OR annoy* OR exasperat* OR irritat* OR nuisance* OR vexat*)) OR (TI (aggravat* OR annoy* OR exasperat* OR irritat* OR nuisance* OR vexat*)))	14,796
**8**	**6 OR 7**	**14,838**
**9**	**5 AND 8**	**329**
10	(MH "Adaptation, Physiological+" OR MH "Stress, Physiological+")	14,512
11	((AB (allosta* OR stress OR stressor*)) OR (TI (allosta* OR stress OR stressor*)))	187,995
12	((AB ((accumulat* or chronic*) AND neuroendocrine)) OR (TI ((accumulat* or chronic*) AND neuroendocrine)))	606
**13**	**OR/10-12**	**198,217**
14	MH "Cardiovascular Diseases+"	668,410
15	((AB (aneurysm* OR arrhythmia* OR atria* OR "blood pressure" OR "blood vessel" OR "blood vessels" OR bradycard* OR "brugada syndrome" OR cardia* OR cardio* OR cerebrovasc* OR "commotio cordis" OR "conduction disturbance" OR "conduction disturbances" OR coronary OR fibrillat* OR dysrhythmia* OR heart OR hypertensi* OR infarct* OR ischemi* OR "long QT syndrome" OR myocard* OR NSTEMI OR parasystole OR "paroxysmal dyspnea" OR "pre-excitation syndrome" OR STEMI OR stroke OR tachycardia* OR vascula* OR vasomotor* OR ventric*)) OR (TI (aneurysm* OR arrhythmia* OR atria* OR "blood pressure" OR "blood vessel" OR "blood vessels" OR bradycard* OR "brugada syndrome" OR cardia* OR cardio* OR cerebrovasc* OR "commotio cordis" OR "conduction disturbance" OR "conduction disturbances" OR coronary OR fibrillat* OR dysrhythmia* OR heart OR hypertensi* OR infarct* OR ischemi* OR "long QT syndrome" OR myocard* OR NSTEMI OR parasystole OR "paroxysmal dyspnea" OR "pre-excitation syndrome" OR STEMI OR stroke OR tachycardia* OR vascula* OR vasomotor* OR ventric*)))	883,895
**16**	**14 OR 15**	**1,089,113**
17	(MH "Amylases" OR MH "Biological Markers+")	93,290

18	((AB (amylase* OR "biochemical marker" OR "biochemical markers" OR "biological marker" OR "biological markers" OR biomarker* OR catecholamine OR "clinical marker" OR "clinical markers" OR diastas* OR "immune marker" OR "immune markers" OR "immunologic marker" OR "immunologic markers" OR "laboratory marker" OR "laboratory markers" OR "serum marker" OR "serum markers" OR "surrogate end point" OR "surrogate end points" OR "surrogate marker" OR "surrogate markers" OR "viral marker" OR "viral markers")) OR (TI (amylase* OR "biochemical marker" OR "biochemical markers" OR "biological marker" OR "biological markers" OR biomarker* OR catecholamine OR "clinical marker" OR "clinical markers" OR diastas* OR "immune marker" OR "immune markers" OR "immunologic marker" OR "immunologic markers" OR "laboratory marker" OR "laboratory markers" OR "serum marker" OR "serum markers" OR "surrogate end point" OR "surrogate end points" OR "surrogate marker" OR "surrogate markers" OR "viral marker" OR "viral markers")))	94,060
19	(MH "Adiponectin" OR MH "Adrenocorticotropic Hormone" OR MH "C-Peptide" OR MH "Glucose Tolerance Test" OR MH "Glycated Hemoglobin" OR MH "Insulin+")	59,908
20	((AB ("ACRP30 protein" OR ACTH OR "adipocyte complement related protein 30 kDa" OR "adipose most abundant gene transcript 1" OR adiponectin OR "adrenocorticotrophic hormone" OR "adrenocorticotropic hormone" OR adrenocorticotropin OR "apM-1 protein" OR corticotrophin OR corticotropin OR corticotropin OR "c-peptide" OR "connecting peptide" OR "fasting plasma glucose" OR "fructated hemoglobin" OR "fructated hemoglobins" OR "glycated A1a-2 hemoglobin" OR "glycated A1a-2 hemoglobins" OR "glycated A1b hemoglobin" OR "glycated A1b hemoglobins" OR "glycated hemoglobin" OR "glycated hemoglobins" OR glycohemoglobin" OR glycohemoglobins" OR "glycosylated A1a-1 hemoglobin" OR "glycosylated A1a-1 hemoglobins" OR "glycosylated A1b hemoglobin" OR "glycosylated A1b hemoglobins" OR "glycosylated hemoglobin" OR "glycosylated hemoglobins" OR "Hb A1" OR "Hb A1a+b" OR "Hb A1a-1" OR "Hb A1a-2" OR "Hb A1b" OR "Hb A1c" OR "Hb A1c" OR "HbA1" OR HbA1c OR "hemoglobin A(1)" OR iletin OR insulin OR novolin OR "OGTT" OR "oral glucose tolerance" OR "urine albumin-to-creatinine ratio")) OR (TI ("ACRP30 protein" OR ACTH OR "adipocyte complement related protein 30 kDa" OR "adipose most abundant gene transcript 1" OR adiponectin OR "adrenocorticotrophic hormone" OR "adrenocorticotropic hormone" OR adrenocorticotropin OR "apM-1 protein" OR corticotrophin OR corticotropin OR corticotropin OR "c-peptide" OR "connecting peptide" OR "fasting plasma glucose" OR "fructated hemoglobin" OR "fructated hemoglobins" OR "glycated A1a-2 hemoglobin" OR "glycated A1a-2 hemoglobins" OR "glycated A1b hemoglobin" OR "glycated A1b hemoglobins" OR "glycated hemoglobin" OR "glycated hemoglobins" OR glycohemoglobin" OR glycohemoglobins" OR "glycosylated A1a-1 hemoglobin"	93,253

	OR "glycosylated A1a-1 hemoglobins" OR "glycosylated A1b hemoglobin" OR "glycosylated A1b hemoglobins" OR "glycosylated hemoglobin" OR "glycosylated hemoglobins" OR "Hb A1" OR "Hb A1a+b" OR "Hb A1a-1" OR "Hb A1a-2" OR "Hb A1b" OR "Hb A1c" OR "Hb A1c" OR "HbA1" OR HbA1c OR "hemoglobin A(1)" OR iletin OR insulin OR novolin OR "OGTT" OR "oral glucose tolerance" OR "urine albumin-to-creatinine ratio")))	
21	(MH "Aldosterone" OR MH "Atrial Natriuretic Factor" OR MH "Brain-Derived Neurotrophic Factor" OR MH "Endothelins" OR MH "Epinephrine+" OR MH "Homocysteine" OR MH "Hydrocortisone" OR MH "Hypertension+" OR MH "Lipids+" OR MH "Norepinephrine" OR MH "Peroxidases+" OR MH "Proteins+")	653,789
22	((AB ("18-Oxocorticosterone" OR "4-α-D-glucan glucanohydrolase" OR "α-Amylase" OR "a-amylase" OR ACTH OR adrenaline OR "adrenocorticotropic hormone" OR aldocorten OR aldocortene OR aldocortin OR aldosterone OR aldosteronum OR "alpha-amylase" OR "alpha ANP" OR adrenomedullin OR arterenol OR "atrial natriuretic factor" OR "atrial natriuretic peptide" OR "atrial natriuretic peptides" OR "atrial pronatriodilatin" OR atriopeptigen OR atriopeptins OR BDNF OR "beta ANP" OR "big endothelin" OR "BNP" OR "brain-derived neurotrophic factor" OR "brain natriuretic peptide" OR cachectin OR "carbohydrate binding protein 35" OR "cardiodilatin precursor" OR "cardionatrin I" OR "cardionatrin IV" OR "cathechol amine*" OR cathecholamin* OR "CBP30" OR "CBP-30" OR "CBP35" OR "CBP-35" OR ceroid OR "coagulation factor I" OR cortef OR corticotropin OR cortifair OR cortifair OR cortisol OR cortril OR cortrophin OR "creactive protein*" OR "c-reactive protein*" OR CRP OR curiculin OR "d dimer" OR electrocortin OR "electronegative low-density lipoprotein" OR elektrocortin OR "endothelin-1" OR "endothelin type 1" OR epicortisol OR epifrin OR epinephrine OR epitrate OR "epsilon binding protein" OR "ET-1" OR "ET1" OR fats OR "fatty acid" OR "fatty acids" OR "fatty alcohol" OR "fatty alcohols" OR "fibrin fragment D" OR fibrinogen OR "galectin 3" OR "gamma ANP" OR glyceride* OR glycolipid* OR "heart rate variability" OR "HL29" OR "HL-29" OR homocysteine OR "HRV" OR hsCRP OR hydrocortisone OR hypertension OR "IgE Binding Protein" OR "IgEBP" OR "L29 Lectin" OR "L-29 Lectin" OR "L30 Lectin" OR "L-30 Lectin" OR "L31" OR "L-31" OR "L34" OR "L-34" OR levarterenol OR levonor OR levonorepinephrine OR levophed OR "LGALS3" OR lipid OR lipids OR lipofuscin OR lipopeptide* OR lipopolysaccharide* OR lipoprotein* OR lyophrin OR "mac 2 antigen" OR "macrophage 2 antigen" OR "medihaler-epi" OR "minimally modified oxidized-LDL" OR "MM-LDL" OR myeloperoxidase OR noradrenaline OR "norepinephrine" OR "oxidized low density lipoprotein" OR "oxidized LDL" OR "ox-LDL" OR OxLDL OR peroxidase OR "prepro ANP" OR "prepro CDD ANF" OR "pro ANF" OR "proANF" OR "proatrial natriuretic factor" OR pronatriodilatin OR pyrocatechinamine* OR pyrocatecholamine OR "Reichstein X" OR sCortisol OR "sICAM-1" OR "soluble intercellular adhesion molecule-1" OR "TNF-α" OR "TNF-a" OR TNFα OR TNFa OR "TNF-alpha" OR TNFalpha OR troponin OR "tumor necrosis factor-alpha")) OR (TI ("18-Oxocorticosterone" OR "4-α-D-glucan glucanohydrolase" OR "α-Amylase" OR "a-amylase" OR ACTH OR adrenaline OR "adrenocorticotropic hormone" OR aldocorten OR aldocortene OR aldocortin OR aldosterone OR aldosteronum OR "alpha-amylase" OR "alpha ANP" OR adrenomedullin OR arterenol OR "atrial natriuretic factor" OR "atrial natriuretic peptide" OR "atrial natriuretic peptides" OR "atrial pronatriodilatin" OR atriopeptigen OR atriopeptins OR BDNF OR "beta ANP" OR "big endothelin" OR "BNP" OR "brain-derived neurotrophic factor" OR "brain natriuretic peptide" OR cachectin OR "carbohydrate binding protein 35" OR "cardiodilatin precursor" OR "cardionatrin I" OR "cardionatrin IV" OR "cathechol amine*" OR cathecholamin* OR "CBP30" OR "CBP-30" OR "CBP35" OR "CBP-35" OR ceroid OR "coagulation factor I" OR cortef OR corticotropin OR cortifair OR cortifair OR cortisol OR cortril OR cortrophin OR "creactive protein*" OR "c-reactive protein*" OR CRP OR curiculin OR "d dimer" OR electrocortin OR "electronegative low-density lipoprotein" OR elektrocortin OR "endothelin-1" OR "endothelin type 1" OR epicortisol OR epifrin OR epinephrine OR epitrate OR "epsilon binding protein" OR "ET-1" OR "ET1" OR fats OR "fatty acid" OR "fatty acids" OR "fatty alcohol" OR "fatty alcohols" OR "fibrin fragment D" OR fibrinogen OR "galectin 3" OR "gamma ANP" OR glyceride* OR glycolipid* OR "heart rate variability" OR "HL29" OR "HL-29" OR homocysteine OR "HRV" OR hsCRP OR hydrocortisone OR hypertension OR "IgE Binding Protein" OR "IgEBP" OR "L29 Lectin" OR "L-29 Lectin" OR "L30 Lectin" OR "L-30 Lectin" OR "L31" OR "L-31" OR "L34" OR "L-34" OR levarterenol OR levonor OR levonorepinephrine OR levophed OR "LGALS3" OR lipid OR lipids OR lipofuscin OR lipopeptide* OR lipopolysaccharide* OR lipoprotein* OR lyophrin OR "mac 2 antigen" OR "macrophage 2 antigen" OR "medihaler-epi" OR "minimally modified oxidized-LDL" OR "MM-LDL" OR myeloperoxidase OR noradrenaline OR "norepinephrine" OR	343,024

	"oxidized low density lipoprotein" OR "oxidized LDL" OR "ox-LDL" OR OxLDL OR peroxidase OR "prepro ANP" OR "prepro CDD ANF" OR "pro ANF" OR "proANF" OR "proatrial natriuretic factor" OR pronatriodilatin OR pyrocatechinamine* OR pyrocatecholamine OR "Reichstein X" OR sCortisol OR "sICAM-1" OR "soluble intercellular adhesion molecule-1" OR "TNF-α" OR "TNF-a" OR TNFα OR TNFa OR "TNF-alpha" OR TNFalpha OR troponin OR "tumor necrosis factor-alpha")))	
23	(MH "Catalase" OR MH "Glutathione Peroxidase" OR MH "Malondialdehyde" OR MH "Nitric Oxide" OR MH "Reactive Oxygen Species+" OR MH "Superoxide Dismutase")	27,887
24	((AB ("8-hydroxy-2'-deoxyguanosine" OR "8OHdG" OR "8-OHdG" OR "active oxygen" OR "advanced oxidation protein products" OR catalase OR "endogenous nitrate vasodilator" OR "endothelium-derived nitric oxide" OR erythrocuprein OR "glutathione lipoperoxidase" OR "glutathione peroxidase" OR hemocuprein OR malonaldehyde OR malondialdehyde OR malonylaldehyde OR malonyldialdehyde OR "Mn-SOD" OR "mononitrogen monoxide" OR "nitric oxide" OR "nitrogen monoxide" OR "oxygen radical" OR "oxygen radicals" OR "pro-oxidant" OR "pro-oxidants" OR propanedial OR "reactive oxygen intermediates" OR "reactive oxygen species" OR "selenoglutathione peroxidase" OR "superoxide dismutase" OR "total antioxidant capacity")) OR (TI ("8-hydroxy-2'-deoxyguanosine" OR "8OHdG" OR "8-OHdG" OR "active oxygen" OR "advanced oxidation protein products" OR catalase OR "endogenous nitrate vasodilator" OR "endothelium-derived nitric oxide" OR erythrocuprein OR "glutathione lipoperoxidase" OR "glutathione peroxidase" OR hemocuprein OR malonaldehyde OR malondialdehyde OR malonylaldehyde OR malonyldialdehyde OR "Mn-SOD" OR "mononitrogen monoxide" OR "nitric oxide" OR "nitrogen monoxide" OR "oxygen radical" OR "oxygen radicals" OR "pro-oxidant" OR "pro-oxidants" OR propanedial OR "reactive oxygen intermediates" OR "reactive oxygen species" OR "selenoglutathione peroxidase" OR "superoxide dismutase" OR "total antioxidant capacity")))	35,580
**25**	**OR/17-24**	**950,748**
26	MH "Cytokines+"	89,900
27	((AB (adipokines OR chemokine* OR cytokine* OR interferon* OR interleukin* OR IL1 OR IL2 OR IL3 OR IL4 OR IL5 OR IL6 OR IL7 OR IL8 OR IL9 OR "IL-1" OR "IL-2" OR "IL-3" OR "IL-4" OR "IL-5" OR "IL-6" OR "IL-7" OR "IL-8" OR "IL-9" OR lymphokine* OR mesenchymal growth factor* OR MGF OR monokine* OR TNF OR "tumor necrosis factor" OR "tumor necrosis factors" OR "tumour necrosis factor" OR "tumour necrosis factors")) OR (TI (adipokines OR chemokine* OR cytokine* OR interferon* OR interleukin* OR IL1 OR IL2 OR IL3 OR IL4 OR IL5 OR IL6 OR IL7 OR IL8 OR IL9 OR "IL-1" OR "IL-2" OR "IL-3" OR "IL-4" OR "IL-5" OR "IL-6" OR "IL-7" OR "IL-8" OR "IL-9" OR lymphokine* OR mesenchymal growth factor* OR MGF OR monokine* OR TNF OR "tumor necrosis factor" OR "tumor necrosis factors" OR "tumour necrosis factor" OR "tumour necrosis factors")))	93,572
**28**	**26 OR 27**	**131,196**

29	MH "Environmental Monitoring+"	6,344
30	((AB ("biological monitor" OR "biological monitors" OR "biological monitoring" OR "biologic monitor" OR "biologic monitors" OR "biologic monitoring" OR biomonitor* OR "bio-monitor" OR "bio-monitors" OR "bio-monitoring")) OR (TI ("biological monitor" OR "biological monitors" OR "biological monitoring" OR "biologic monitor" OR "biologic monitors" OR "biologic monitoring" OR biomonitor* OR "bio-monitor" OR "bio-monitors" OR "bio-monitoring")))	949
31	(MH "Hair Analysis" OR MH "Hematologic Tests+" OR MH "Urinalysis" OR ((MH "Blood+" OR MH "Feces+" OR MH "Hair+" OR MH "Milk, Human+" OR MH "Saliva" OR MH "Sputum" OR MH "Urine") AND ((AB (analy* OR assay* OR concentration* OR level* OR sampl* OR test*)) OR (TI (analy* OR assay* OR concentration* OR level* OR sampl* OR test*)))))	114,104
32	(((AB ("breast milk" OR blood OR faecal OR faeces OR fecal OR feces OR hair OR haematolog* OR hematolog* OR "human milk" OR plasma OR saliv* OR sera OR serolog* OR serum OR sputum* OR stool OR urina* OR urine*)) OR (TI ("breast milk" OR blood OR faecal OR faeces OR fecal OR feces OR hair OR haematolog* OR hematolog* OR "human milk" OR plasma OR saliv* OR sera OR serolog* OR serum OR sputum* OR stool OR urina* OR urine*))) AND ((AB (analy* OR concentration* OR level* OR sampl* OR test*)) OR (TI (analy* OR concentration* OR level* OR sampl* OR test*))))	408,898
**33**	**OR/29-32**	**468,174**
**34**	**13 OR 16 OR 25 OR 28 OR 33**	**2,027,262**
**35**	**9 AND 34**	**108**
36	Language: English	
**37**	**35 AND 36**	**105**
38	((MH "Clinical Research+" OR MH "Clinical Trials+" OR MH "Comparative Studies+" OR MH "Multicenter Studies" OR (AB (compara* OR compare* OR compari* OR placebo OR random*)) OR (TI (compara* OR compare* OR compari* OR placebo OR random* OR trial*)) OR (AB (case AND (control OR report* OR series OR studies OR study))) OR (TI (case AND (control OR report* OR series OR studies OR study))) OR (((AB (clinical OR cohort OR crossover OR "cross-over" OR "cross-sectional" OR multicenter)) OR (TI (clinical OR cohort OR crossover OR "cross-over" OR "cross-sectional" OR multicenter))) AND ((AB (studies OR study OR trial*)) OR (TI (studies OR study OR trial*))))) OR (MH "Meta Analysis" OR MH "Literature Review+" OR MH "Systematic Review" OR (AB (metaanalys* OR meta-analys* OR "meta analys*" OR review OR "systematic review")) OR (TI (metaanalys* OR meta-analys* OR "meta analys*" OR review OR "systematic review")) OR ((TI (meta OR systematic*) AND (overview* OR review* OR synthesis*)))))	
**39**	**37 AND 38**	**78**

Table S2. List of ineligible studies

Author / Citation #	Title	Reason for Exclusion
Melamed [47]	Industrial Noise Exposure and Risk Factors for Cardiovascular Disease: Findings from the CORDIS Study	Duplicate population of Melamed 1997
Eriksson [48]	Aircraft noise and incidence of hypertension	Duplicate population of Eriksson 2010
Eriksson [49]	Aircraft noise and incidence of hypertension--gender specific effects	Exclusion reason: Wrong exposure;
Hahad [50]	Midregional pro atrial natriuretic peptide: a novel important biomarker for noise annoyance-induced cardiovascular morbidity and mortality?	Duplicate of Hahad 2021
Lercher [51]	Hypotension and environmental noise: a replication study	Exclusion reason: Wrong exposure;
Aasvang [52]	Burden of disease due to transportation noise in the Nordic countries	Exclusion reason: Wrong exposure;
Abbasi [53]	Combined effects of noise and air temperature on human neurophysiological responses in a simulated indoor environment	Exclusion reason: Wrong exposure;
ACTRN12619000027112 [54]	Assessing the health effects of six months of simulated wind farm infrasound: a community-based randomised controlled trial	Exclusion reason: Study design;
Agarwal [55]	Road traffic noise, annoyance and community health survey - a case study for an Indian city	Exclusion reason: Wrong exposure;
Al-Harthy [56]	Assessment of noise levels and induced annoyance in nearby residential areas of an airport region in Oman	Exclusion reason: Wrong exposure;

**Author / Citation #**	**Title**	**Reason for Exclusion**
Al-Mutairi [57]	Traffic-generated noise pollution: exposure of road users and populations in Metropolitan Kuwait	Exclusion reason: Wrong exposure;
Alkhalawi [58]	Residential traffic noise exposure and headaches: Results from the population-based heinz nixdorf recall study.	Exclusion reason: Wrong outcome;
Amoatey [59]	Exposure assessment to road traffic noise levels and health effects in an arid urban area	Exclusion reason: Wrong outcome;
Amoatey [60]	Effect of ambient noise on indoor environments in a health care facility in Oman	Exclusion reason: Wrong exposure;
Amoatey [25]	Contribution of outdoor noise-induced health risk in schools located in urbanized arid country	Exclusion reason: Wrong exposure;
Babisch [61]	Health status as a potential effect modifier of the relation between noise annoyance and incidence of ischaemic heart disease	Exclusion reason: Wrong outcome;
Babisch [62]	Traffic noise and risk of myocardial infarction	Exclusion reason: Wrong outcome;
Babisch [63]	Exposure modifiers of the relationships of transportation noise with high blood pressure and noise annoyance	Exclusion reason: Wrong exposure;
Babisch [64]	Road traffic noise and hypertension - Results from the kora study	Exclusion reason: Abstract only;
Baudin [65]	Self-rated health status in relation to aircraft noise exposure, noise annoyance or noise sensitivity: the results of a cross-sectional study in France	Exclusion reason: Wrong outcome;
Baudin [66]	Self-rated health status in relation to aircraft noise exposure, noise annoyance or noise sensitivity: the results of a cross-sectional study in France.	Duplicate
Belojevic [67]	Prevalence of Arterial Hypertension and Myocardial Infarction in Relation to Subjective Ratings of Traffic Noise Exposure	Exclusion reason: Wrong outcome;
Belojević [68]	Nighttime road-traffic noise and arterial hypertension in an urban population	Exclusion reason: Wrong exposure;

Author / Citation #	Title	Reason for Exclusion
Belojevic [69]	Cardiovascular effects of environmental noise: research in Serbia	Exclusion reason: Study design;
Beutel [70]	Noise Annoyance Is Associated with Depression and Anxiety in the General Population- The Contribution of Aircraft Noise	Exclusion reason: Wrong outcome;
Bosch [71]	The Influence of annoyance due to aircraft noise on renal hemodynamic	Exclusion reason: Abstract only;
Bosch [72]	The influence of annoyance due to aircraft noise on renal hemodynamic	Duplicate
Bosch [73]	Annoyance due to noise triggers vascular changes in the renal hemodynamic	Exclusion reason: Abstract only;
Bosch [74]	The influence of aircraft noise exposure on the systemic and renal haemodynamics	Exclusion reason: Wrong exposure;
Bräuner [75]	Long-term wind turbine noise exposure and incidence of myocardial infarction in the Danish nurse cohort	Exclusion reason: Wrong outcome;
Byers [76]	Efficacy of a music intervention on noise annoyance and selected physiologic variables in cardiac surgery patients with high and low noise sensitivity.	Exclusion reason: Wrong exposure;
Byers [77]	Effect of a music intervention on noise annoyance, heart rate, and blood pressure in cardiac surgery patients	Exclusion reason: Wrong exposure;
Carugno [78]	Effects of aircraft noise on annoyance, sleep disorders, and blood pressure among adult residents near the Orio al Serio International Airport (BGY), Italy	Exclusion reason: Wrong exposure;
Chakraborty [79]	Cumulative Noise Exposure and Perceived Effects: A Comparative Study Among Different Occupational Groups in Kolkata	Exclusion reason: Wrong exposure;
Dias [80]	Noise annoyance, sociodemographic and health patterns, and neighborhood perceptions in a brazilian metropolis: Bh health study	Exclusion reason: Wrong exposure;
Dopico [81]	How Do Road Traffic Noise and Residential Greenness Correlate with Noise Annoyance and Long-Term Stress? Protocol and Pilot Study for a Large Field Survey with a Cross-Sectional Design	Exclusion reason: Wrong exposure;

Author / Citation #	Title	Reason for Exclusion
DRKS00011362 [82]	Fragile sleep under Aircraft Noise and Circadian Shift - Examination about the influence of air traffic noise on the vulnerable sleep induced by shift of circadian rhythm	Exclusion reason: Study design;
Dzhambov [83]	Association between community noise and adiposity in patients with cardiovascular disease	Exclusion reason: Wrong exposure;
Eriksson [84]	Traffic noise and cardiovascular health in Sweden: the roadside study	Exclusion reason: Wrong exposure;
Eriksson [85]	Burden of disease from road traffic and railway noise ‚Äì a quantification of healthy life years lost in Sweden	Exclusion reason: Wrong exposure;
Eze [86]	Transportation noise exposure, noise annoyance and respiratory health in adults: A repeated-measures study	Exclusion reason: Wrong outcome;
Eze [87]	Incidence of depression in relation to transportation noise exposure and noise annoyance in the SAPALDIA study	Exclusion reason: Wrong outcome;
Farooqi [88]	Assessment of noise pollution and its effects on human health in industrial hub of Pakistan	Exclusion reason: Wrong exposure;
Farooqi [89]	Urban noise assessment and its non auditory health effects on the residents of Chiniot and Jhang, Punjab, Pakistan	Exclusion reason: Wrong exposure;
Flanagan [90]	Health impact assessment of road traffic noise exposure based on different densification scenarios in Malm√∂, Sweden	Exclusion reason: Wrong exposure;
Fooladi [91]	Involuntary and persistent environmental noise influences health and hearing in Beirut, Lebanon	Exclusion reason: Wrong exposure;
Foraster [92]	Impact of long- and shorter-term annoyance to transportation noise at home on physical inactivity	Exclusion reason: Abstract only;
Fyhri [93]	Noise, sleep and poor health: Modeling the relationship between road traffic noise and cardiovascular problems	Exclusion reason: Wrong outcome;
Haines [94]	Chronic aircraft noise exposure, stress responses, mental health and cognitive performance in school children	Exclusion reason: Wrong outcome;

**Author / Citation #**	**Title**	**Reason for Exclusion**
Hauptvogel [95]	Being a Fair Neighbor,ÄîTowards a Psychometric Inventory to Assess Fairness-Related Perceptions of Airports by Residents,ÄîDevelopment and Validation of the Aircraft Noise-Related Fairness Inventory (fAIR-In)	Exclusion reason: Wrong exposure;
IRCT201411304443 N16 [96]	music therapy and mechanical ventilation	Exclusion reason: Wrong exposure;
Ising [97]	Annoyance and health risk caused by military low-altitude flight noise	Exclusion reason: Wrong exposure;
Ising [98]	Low frequency noise and stress: bronchitis and cortisol in children exposed chronically to traffic noise and exhaust fumes	Exclusion reason: Wrong exposure;
Itzkowitz [99]	Aircraft noise and cardiovascular morbidity and mortality near Heathrow Airport: A case-crossover study	Exclusion reason: Wrong exposure;
Jarup [100]	Hypertension and Exposure to Noise near Airports (HYENA): study design and noise exposure assessment	Exclusion reason: Wrong exposure;
Jensen [101]	Neighbour and traffic noise annoyance: a nationwide study of associated mental health and perceived stress	Exclusion reason: Wrong outcome;
Jensen [102]	Neighbour noise annoyance is associated with various mental and physical health symptoms: results from a nationwide study among individuals living in multi-storey housing	Exclusion reason: Wrong outcome;
Kasicka-Jonderko [103]	Extracirculatory effects of noise of various frequency spectra in humans--effect of pink and blue noise on gastric myoelectrical activity and gastrointestinal passage of nutrients	Exclusion reason: Wrong exposure;
Kooshanfar [104]	Sources of noise and their effects on nurses in intensive care units: A cross sectional study	Exclusion reason: Wrong exposure;
Lamb [105]	A longitudinal investigation of work environment stressors on the performance and wellbeing of office workers.	Exclusion reason: Wrong outcome;
Lusk [106]	Chronic effects of workplace noise on blood pressure and heart rate	Exclusion reason: Wrong exposure;

Author / Citation #	Title	Reason for Exclusion
Mabote [107]	Effect of music and noise on haemodynamics in patients with heart failure. HeartCycle (FP7-216695) European union 7th framework programme	Exclusion reason: Abstract only;
Malinauskiene [108]	The associations between urban traffic emissions on myocardial infarction risk	Exclusion reason: Abstract only;
Marshall [109]	The Health Effects of 72 Hours of Simulated Wind Turbine Infrasound: A Double-Blind Randomized Crossover Study in Noise-Sensitive, Healthy Adults	Exclusion reason: Wrong exposure;
Melamed [110]	Noise exposure, noise annoyance and their relation to psychological distress, accident and sickness absence among blue-collar workers--the Cordis Study.	Exclusion reason: Wrong outcome;
Méline [111]	Transportation noise and annoyance related to road traffic in the French RECORD study	Exclusion reason: Wrong exposure;
Moller [112]	Physiological and psychological effects of infrasound on humans	Exclusion reason: Wrong exposure;
Morrison [113]	Noise, stress, and annoyance in a pediatric intensive care unit	Exclusion reason: Wrong exposure;
NCT04809545 [114]	Effect of Soundscape on People With Dementia	Exclusion reason: Study design;
NCT05319262 [115]	Metabolic and Cognitive Consequences of Noise-induced Sleep Disturbance	Exclusion reason: Study design;
NCT05611619 [116]	Low Particle Emission and Low Noise Tyres	Exclusion reason: Study design;
Nguyen [117]	Effects of Changes in Acoustic and Non-Acoustic Factors on Public Health and Reactions: Follow-Up Surveys in the Vicinity of the Hanoi Noi Bai International Airport	Exclusion reason: Wrong exposure;
Nitschke [118]	The link between noise perception and quality of life in South Australia	Exclusion reason: Wrong exposure;
Okokon [119]	Road-traffic noise: Annoyance, risk perception, and noise sensitivity in the finnish adult population	Exclusion reason: Wrong exposure;
Okokon [120]	Traffic noise, noise annoyance and psychotropic medication use	Exclusion reason: Wrong outcome;
Ouis [121]	Annoyance caused by exposure to road traffic noise: An update	Exclusion reason: Study design;

Author / Citation #	Title	Reason for Exclusion
Pawlaczyk-Łuszczyńska [122]	Response to noise emitted by wind farms in people living in nearby areas	Exclusion reason: Wrong exposure;
Poulsen [123]	Short-term nighttime wind turbine noise and cardiovascular events: A nationwide case-crossover study from Denmark	Exclusion reason: Abstract only;
Poulsen [124]	Long-term exposure to wind turbine noise and redemption of antihypertensive medication: A nationwide cohort study	Exclusion reason: Wrong exposure;
Poulsen [123]	Long-term exposure to wind turbine noise at night and risk for diabetes: A nationwide cohort study	Exclusion reason: Wrong exposure;
Poulsen [125]	Long-Term Exposure to Wind Turbine Noise and Risk for Myocardial Infarction and Stroke: A Nationwide Cohort Study	Exclusion reason: Wrong exposure;
Pulles [126]	Adverse effects of environmental noise on health: An interdisciplinary approach	Exclusion reason: Wrong exposure;
Radun [127]	Speech is special: The stress effects of speech, noise, and silence during tasks requiring concentration	Exclusion reason: Wrong exposure;
Rahman [128]	Perceived Noise Pollution and Self-Reported Health Status among Adult Population of Bangladesh	Exclusion reason: Wrong exposure;
Riedel [129]	Home as a Place of Noise Control for the Elderly? A Cross-Sectional Study on Potential Mediating Effects and Associations between Road Traffic Noise Exposure, Access to a Quiet Side, Dwelling-Related Green and Noise Annoyance	Exclusion reason: Wrong exposure;
Riedel [130]	Urban road traffic noise and noise annoyance-a study on perceived noise control and its value among the elderly	Exclusion reason: Wrong exposure;
Rojek [131]	Nighttime aircraft noise effect on blood pressure profile	Exclusion reason: Abstract only;
Rojek [132]	Environmental noise and arterial stiffness in the context of hypertension	Exclusion reason: Abstract only;
Rojek [133]	The relation of nocturnal exposure to aircraft noise and aircraft noise,Äìinduced insomnia with blood pressure	Exclusion reason: Wrong outcome;

**Author / Citation #**	**Title**	**Reason for Exclusion**
Sandrock [134]	Mental strain and annoyance during cognitive performance in different traffic noise conditions.	Exclusion reason: Wrong exposure;
Schmidt [135]	Nighttime aircraft noise impairs endothelial function and increases blood pressure in patients with or at high risk for coronary artery disease	Exclusion reason: Wrong exposure;
Schmidt [136]	Nighttime aircraft noise impairs endothelial function and increases blood pressure in patients with or at high risk for coronary artery disease	Exclusion reason: Wrong exposure;
Schmidt [137]	The impact of aircraft noise on vascular and cardiac function in relation to noise event number: a randomized trial	Exclusion reason: Wrong exposure;
Schreckenberg [138]	Aircraft noise and quality of life around Frankfurt Airport	Exclusion reason: Wrong outcome;
Schreckenberg [139]	The associations between noise sensitivity, reported physical and mental health, perceived environmental quality, and noise annoyance.	Exclusion reason: Wrong exposure;
Selander [140]	Long-term exposure to road traffic noise and myocardial infarction	Exclusion reason: Wrong outcome;
Şentop Dümen [141]	Noise annoyance during COVID-19 lockdown: A research of public opinion before and during the pandemic	Exclusion reason: Wrong outcome;
Sieber [44]	Comparison of sensitivity and annoyance to road traffic and community noise between a South African and a Swiss population sample	Exclusion reason: Wrong exposure;
Smith [142]	Self-reported sleep disturbance by aircraft noise around Atlanta airport	Exclusion reason: Abstract only;
Sobotova [143]	Community response to environmental noise and the impact on cardiovascular risk score	Exclusion reason: Wrong exposure;
Stamatin [144]	The impact of high noise exposure on textile workers health-a retrospective 3 years study in a textile factory	Exclusion reason: Abstract only;
Stansfeld [145]	Noise sensitivity and future risk of illness and mortality	Exclusion reason: Wrong outcome;

Author / Citation #	Title	Reason for Exclusion
Stansfeld [146]	Road traffic noise, noise sensitivity, noise annoyance, psychological and physical health and mortality	Exclusion reason: Wrong outcome;
Thiesse [147]	Transportation noise impairs cardiovascular function without altering sleep: The importance of autonomic arousals	Exclusion reason: Wrong exposure;
Tobollik [148]	Burden of Disease Due to Traffic Noise in Germany	Exclusion reason: Wrong outcome;
Tsoi [149]	The co-benefits of electric mobility in reducing traffic noise and chemical air pollution: Insights from a transit-oriented city	Exclusion reason: Wrong exposure;
Turunen [150]	Self-reported health in the vicinity of five wind power production areas in Finland	Exclusion reason: Wrong exposure;
vanDijk [151]	Non-auditory effects of noise in industry. VI. A final field study in industry	Exclusion reason: Wrong exposure;
vanDijk [89]	Non-auditory effects of noise in industry. V. A field study in a shipyard	Exclusion reason: Wrong exposure;
VanDijk [152]	Non-auditory effects of noise in industry. VI. A final study in industry	Exclusion reason: Wrong exposure;
Wallas [153]	Noise exposure and childhood asthma up to adolescence	Exclusion reason: Wrong exposure;
Wallner [154]	Evaluating an 80 Hz tonal noise from a hydropower plant.	Exclusion reason: Wrong exposure;
Wang [155]	Associations between personal noise exposure and heart rate variability were modified by obesity and PM(2.5): The study among obese and normal-weight adults (SONA)	Exclusion reason: Wrong exposure;
Waye [156]	Low frequency noise enhances cortisol among noise sensitive subjects during work performance	Exclusion reason: Wrong exposure;
Webb [157]	Effects of hospital noise on heart rate variability of acutely ill adults.	Exclusion reason: Study design;
Willich [158]	Noise burden and the risk of myocardial infarction	Exclusion reason: Wrong outcome;
Wojciechowska [159]	Impact of reduction in aircraft noise exposure on blood pressure and arterial stiffness during	Exclusion reason: Abstract only;

**Author / Citation #**	**Title**	**Reason for Exclusion**
	COVID-19 pandemic: Cross-sectional and longitudinal study	
Wojciechowska [160]	Blood Pressure and Arterial Stiffness in Association With Aircraft Noise Exposure:Long-Term Observation and Potential Effect of COVID-19 Lockdown	Exclusion reason: Wrong exposure;
Wojciechowska [161]	NOISE POLLUTION AND ARTERIAL HYPERTENSION	Exclusion reason: Study design;
Wothge [162]	The Combined Effects of Aircraft and Road Traffic Noise and Aircraft and Railway Noise on Noise Annoyance-An Analysis in the Context of the Joint Research Initiative NORAH	Exclusion reason: Wrong exposure;
Yang [163]	Road Traffic Noise, Air Pollutants, and the Prevalence of Cardiovascular Disease in Taichung, Taiwan	Exclusion reason: Wrong exposure;
Zijlema [164]	Noise and somatic symptoms: A role for personality traits?	Exclusion reason: Wrong outcome;
Zijlema [43]	The LifeLines Cohort Study: a resource providing new opportunities for environmental epidemiology	Exclusion reason: Study design;

References

1. Sivakumaran, K.; Ritonja, J.A.; Waseem, H.; AlShenaiber, L.; Morgan, E.; Ahmadi, S.A.; Denning, A.; Michaud, D.; Morgan, R.L. Impact of Noise Exposure on Risk of Developing Stress-Related Metabolic Effects: A Systematic Review and Meta-Analysis. *Noise Health* **2022**, *24*, 215–230. https://doi.org/10.4103/nah.nah_21_22. PMID: 36537446; PMCID: PMC10088431.
2. Sivakumaran, K.; Ritonja, J.A.; Waseem, H.; AlShenaiber, L.; Morgan, E.; Ahmadi, S.A.; Denning, A.; Michaud, D.S.; Morgan, R.L. Impact of Noise Exposure on Risk of Developing Stress-Related Health Effects Related to the Cardiovascular System: A Systematic Review and Meta-Analysis. *Noise Health* **2022**, *24*, 107–129. https://doi.org/10.4103/nah.nah_83_21.
3. Sivakumaran, K.; Ritonja, J.A.; Waseem, H.; AlShenaiber, L.; Morgan, E.; Ahmadi, S.A.; Denning, A.; Michaud, D.S.; Morgan, R.L. Impact of Noise Exposure on Risk of Developing Stress-Related Obstetric Health Effects: A Systematic Review and Meta-Analysis. *Noise Health* **2022**, *24*, 137–144. https://doi.org/10.4103/nah.nah_22_22.
4. Basner, M.; McGuire, S. WHO environmental noise guidelines for the European region: A systematic review on environmental noise and effects on sleep. *Int. J. Environ. Res. Public Health* **2018**, *15*, 519.
5. van Kempen, E.; Casas, M.; Pershagen, G.; Foraster, M. WHO environmental noise guidelines for the European region: A systematic review on environmental noise and cardiovascular and metabolic effects: A summary. *Int. J. Environ. Res. Public Health* **2018**, *15*, 379.
6. World Health Organization 2011. Burden of Disease from Environmental Noise. Available online: https://iris.who.int/handle/10665/326424 (25 June 2024).
7. Guski, R.; Schreckenberg, D.; Schuemer, R. WHO Environmental Noise Guidelines for the European Region: A Systematic Review on Environmental Noise and Annoyance. *Int. J. Environ. Res. Public Health* **2017**, *14*, 1539.
8. Babisch, W. The noise/stress concept, risk assessment and research needs. *Noise Health* **2002**, *4*, 1–11.
9. McEwen, B.; Lasley, E.N. Allostatic load: When protection gives way to damage. *Adv. Mind Body Med*. **2003**, *19*, 28–33.
10. Anisman, H. *Stress and Your Health: From Vulnerability to Resilience*; John Wiley & Sons, Ltd.: West Sussex, UK, 2015.
11. Health Canada. Guidance for Evaluating Human Health Impacts in Environmental Assessment: Noise. Her Majesty the Queen in Right of Canada, as Represented by the Minister of Health.. 2023. Available online: https://publications.gc.ca/site/eng/9.832514/publication.html (accessed on 25 June 2024).
12. Michaud, D.S.; Bly, S.H.P.; Keith, S.E. Using a change in percent highly annoyed with noise as a potential health effect measure for projects under the Canadian Environmental Assessment Act. *Can. Acoust.* **2008**, *36*, 13–28.
13. Charalampous, P.; Polinder, S.; Wothge, J.; von der Lippe, E.; Haagsma, J.A. A systematic literature review of disability weights measurement studies: Evolution of methodological choices. *Arch. Public Health*. **2022**, *80*, 91. https://doi.org/10.1186/s13690-022-00860-z. PMID: 35331325; PMCID: PMC8944058.
14. World Health Organization. *Basic Documents: Forty-Ninth Edition (Including Amendments Adopted up to 31 May 2019);* World Health Organization: Geneva, Switzerland, 2020.
15. World Health Organization. *Environmental Noise Guidelines for the European Region*; WHO Regional Office for Europe: Copenhagen, Denmark, 2018.
16. Gong, X.; Fenech, B.; Blackmore, C.; Chen, Y.; Rodgers, G.; Gulliver, J.; Hansell, A.L. Association between noise annoyance and mental health outcomes: A systematic review and meta-analysis. *Int. J. Environ. Res. Public Health* **2022**, *19*, 2696. https://doi.org/10.3390/ijerph19052696.
17. Page, M.J.; McKenzie, J.E.; Bossuyt, P.M.; Boutron, I.; Hoffmann, T.C.; Mulrow, C.D.; Shamseer, L.; Tetzlaff, J.M.; Akl, E.A.; Brennan, S.E.; et al. The PRISMA 2020 statement: An updated guideline for reporting systematic reviews. *BMJ* **2021**, *372*, n71. https://doi.org/10.1136/bmj.n71.
18. Clark, C.; Gjestland, T.; Lavia, L.; Notley, H.; Michaud, D.; Morinaga, M. Revising ISO/TS 15666—The noise annoyance standard. In Proceedings of the International Commission on Biological Effects of Noise, Stockholm, Sweden, 14–17 June 2021.
19. Higgins, J.; Morgan, R.; Rooney, A.; Taylor, K.; Thayer, K.; Silva, R.; Lemeris, C.; Akl, A.; Arroyave, W.; Bateson, T.; et al. Risk of Bias In Non-randomized Studies-of Exposure (ROBINS-E). Launch version, 20 June 2023. Available online: https://www.riskofbias.info/welcome/robins-e-tool (accessed on 25 June 2024).
20. Guyatt, G.; Oxman, A.D.; Akl, E.A.; Kunz, R.; Vist, G.; Brozek, J.; Norris, S.; Falck-Ytter, Y.; Glasziou, P.; DeBeer, H.; et al. GRADE guidelines: 1. Introduction-GRADE evidence profiles and summary of findings tables. *J. Clin. Epidemiol*. **2011**, *64*, 383–394. https://doi.org/10.1016/j.jclinepi.2010.04.026.

21. Melamed, S.; Froom, P.; Kristal-Boneh, E.; Gofer, D.; Ribak, J. Industrial noise exposure, noise annoyance, and serum lipid levels in blue-collar workers—The CORDIS Study. *Arch. Environ. Health* **1997**, *52*, 292–298; Erratum in *Arch. Environ. Health* **1997**, *52*, 389. https://doi.org/10.1080/00039899709602201.
22. Arvidsson, O.; Lindvall, T. Subjective annoyance from noise compared with some directly measurable effects. *Arch. Environ. Health* **1978**, *33*, 159–166. https://doi.org/10.1080/00039896.1978.10667328.
23. Badihian, N.; Riahi, R.; Qorbani, M.; Motlagh, M.E.; Heshmat, R.; Kelishadi, R. The associations between noise annoyance and psychological distress with blood pressure in children and adolescents: The CASPIAN-V Study. *J. Clin. Hypertens.* **2020**, *22*, 1434–1441. https://doi.org/10.1111/jch.13946.
24. Wallas, A.; Eriksson, C.; Gruzieva, O.; Lind, T.; Pyko, A.; Sjöström, M.; Ögren, M.; Pershagen, G. Road traffic noise and determinants of saliva cortisol levels among adolescents. *Int. J. Hyg. Environ. Health* **2018**, *221*, 276–282. https://doi.org/10.1016/j.ijheh.2017.11.003.
25. Fields, J.M.; Jong, R.G.; Gjestland, T.; Flindell, I.H.; Job, R.F.S.; Kurra, S.; Lercher, P.; Vallet, M.; Yano, T.; Guski, R.; et al. Standardized general-purpose noise reaction questions for community noise surveys: Research and a recommendation. *J. Sound Vib.* **2001**, *242*, 641–679. https://doi.org/10.1006/jsvi.2000.3384.
26. Alimohammadi, I.; Kanrash, F.A.; Vosoughi, S.; Shekaftik, S.O.; Rahmani, K.; Chalak, M.H.; Anbari, M. Relationship between Noise Annoyance and High Blood Pressure in Workers Exposed to Chronic Noise Among the Workers of an Automotive Industry. *Indian J. Occup. Environ. Med*. **2020**, *24*, 153–156. https://doi.org/10.4103/ijoem.IJOEM_259_19.
27. Babisch, W.; Fromme, H.; Beyer, A.; Ising, H. Increased catecholamine levels in urine in subjects exposed to road traffic noise: The role of stress hormones in noise research. *Environ. Int*. **2001**, *26*, 475–481. https://doi.org/10.1016/s0160-4120(01)00030-7.
28. Babisch, W.; Pershagen, G.; Selander, J.; Houthuijs, D.; Breugelmans, O.; Cadum, E.; Vigna-Taglianti, F.; Katsouyanni, K.; Haralabidis, A.S.; Dimakopoulou, K.; et al. Noise annoyance—A modifier of the association between noise level and cardiovascular health? *Sci. Total Environ*. **2013**, *452 – 453*, 50 – 57. https://doi.org/10.1016/j.scitotenv.2013.02.034.
29. Baudin, C.; Lefevre, M.; Babisch, W.; Cadum, E.; Champelovier, P.; Dimakopoulou, K.; Houthuijs, D.; Lambert, J.; Laumon, B.; Pershagen, G.; et al. The role of aircraft noise annoyance and noise sensitivity in the association between aircraft noise levels and hypertension risk: Results of a pooled analysis from seven European countries. *Environ. Res*. **2020**, *191*, 110179. https://doi.org/10.1016/j.envres.2020.110179.
30. Dzhambov, A.M.; Tokmakova, M.P.; Gatseva, P.D.; Zdravkov, N.G.; Gencheva, D.G.; Ivanova, N.G.; Karastanev, K.I.; Vladeva, S.V.; Donchev, A.T.; Dermendzhiev, S.M. Community Noise Exposure and its Effect on Blood Pressure and Renal Function in Patients with Hypertension and Cardiovascular Disease. *Folia Med.* **2017**, *59*, 344–356. https://doi.org/10.1515/folmed-2017-0045.
31. Fyhri, A.; Klaeboe, R. Road traffic noise, sensitivity, annoyance and self-reported health—A structural equation model exercise. *Environ. Int*. **2009**, *35*, 91–97. https://doi.org/10.1016/j.envint.2008.08.006.
32. Gallasch, E.; Raggam, R.B.; Cik, M.; Rabensteiner, J.; Lackner, A.; Piber, B.; Marth, E. Road and rail traffic noise induce comparable extra-aural effects as revealed during a short-term memory test. *Noise Health* **2016**, *18*, 206–213. https://doi.org/10.4103/1463-1741.189243.
33. Hahad, O.; Beutel, M.; Gori, T.; Schulz, A.; Blettner, M.; Pfeiffer, N.; Rostock, T.; Lackner, K.; Sørensen, M.; Prochaska, J.H.; et al. Annoyance to different noise sources is associated with atrial fibrillation in the Gutenberg Health Study. *Int. J. Cardiol*. **2018**, *264*, 79–84. https://doi.org/10.1016/j.ijcard.2018.03.126.
34. Hahad, O.; Wild, P.S.; Prochaska, J.H.; Schulz, A.; Lackner, K.J.; Pfeiffer, N.; Schmidtmann, I.; Michal, M.; Beutel, M.; Daiber, A.; et al. Midregional pro atrial natriuretic peptide: A novel important biomarker for noise annoyance-induced cardiovascular morbidity and mortality? *Clin. Res. Cardiol*. **2021**, *110*, 29–39. https://doi.org/10.1007/s00392-020-01645-6.
35. Huang, Z.; Aarab, G.; Ravesloot, M.J.L.; de Vries, N.; Hilgevoord, A.A.J.; Lobbezoo, F. The Annoying Nature of Snoring Sounds Is Not Only about Intensity: A Pilot Study on Exposed Test Subjects. *J. Clin. Med*. **2023**, *12*, 2630. https://doi.org/10.3390/jcm12072630.
36. Kannenkeril, D.; Jung, S.; Ott, C.; Striepe, K.; Kolwelter, J.; Schmieder, R.E.; Bosch, A. Association of Noise Annoyance with Measured Renal Hemodynamic Changes. *Kidney Blood Press Res*. **2021**, *46*, 323–330. https://doi.org/10.1159/000515527.
37. Lercher, P.; Hörtnagl, J.; Kofler, W.W. Work noise annoyance and blood pressure: Combined effects with stressful working conditions. *Int. Arch. Occup. Environ Health* **1993**, *65*, 23–28. https://doi.org/10.1007/BF00586054.
38. Michaud, D.S.; Feder, K.; Keith, S.E.; Voicescu, S.A.; Marro, L.; Than, J.; Guay, M.; Denning, A.; Bower, T.; Villeneuve, P.J.; et al. Self-reported and measured stress related responses associated with exposure to wind turbine noise. *J. Acoust. Soc. Am*. **2016**, *139*, 1467–1479. https://doi.org/10.1121/1.4942402.

39. Niemann, H.; Bonnefoy, X.; Braubach, M.; Hecht, K.; Maschke, C.; Rodrigues, C.; Robbel, N. Noise-induced annoyance and morbidity results from the pan-European LARES study. *Noise Health* **2006**, *8*, 63–79. https://doi.org/10.4103/1463-1741.33537.
40. Petri, D.; Licitra, G.; Vigotti, M.A.; Fredianelli, L. Effects of exposure to road, railway, airport and recreational noise on blood pressure and hypertension. *Int. J. Environ. Res. Public Health* **2021**, *18*, 9145. https://doi.org/10.3390/ijerph18179145.
41. Pitchika, A.; Hampel, R.; Wolf, K.; Kraus, U.; Cyrys, J.; Babisch, W.; Peters, A.; Schneider, A. Long-term associations of modeled and self-reported measures of exposure to air pollution and noise at residence on prevalent hypertension and blood pressure. *Sci. Total Environ.* **2017**, *593 – 594*, 337 – 346. https://doi.org/10.1016/j.scitotenv.2017.03.156.
42. Rojek, M.; Rajzer, M.W.; Wojciechowska, W.; Drozdz, T.; Skalski, P.; Pizon, T.; Januszewicz, A.; Czarnecka, D. Relationship among long-term aircraft noise exposure, blood pressure profile, and arterial stiffness. *J. Hypertens.* **2019**, *37*, 1350–1358. https://doi.org/10.1097/HJH.0000000000002060.
43. Stošić, L.; Stojanović, D.; Lazarević, K. The relationship between community noise and arterial hypertension in the population of the city of Niš. *Acta Fac. Medicae Naissensis* **2022**, *39*, 484–490. https://doi.org/10.5937/afmnai39-34187.
44. Vandasova, Z.; Vencálek, O.; Puklová, V. Specific and combined subjective responses to noise and their association with cardiovascular diseases. *Noise Health* **2016**, *18*, 338–346. https://doi.org/10.4103/1463-1741.195800.
45. Michaud, D.S.; Keith, S.E.; McMurchy, D. Annoyance and disturbance of daily activities from road traffic noise in Canada. *J. Acoust. Soc. Am.* **2008**, *123*, 784–792. https://doi.org/10.1121/1.2821984.
46. Kirschbaum, C.; Hellhammer, D.H. Noise and Stress-Salivary cortisol as a non-invasive measure of allostatic load. *Noise Health* **1999**, *1*, 57–66.

[47] S. Melamed, E. Kristal-Boneh, and P. Froom, "Industrial Noise Exposure and Risk Factors for Cardiovascular Disease: Findings from the CORDIS Study," *Noise Health*, vol. 1, no. 4, pp. 49–56, 1999.

[48] C. Eriksson, M. Rosenlund, G. Pershagen, A. Hilding, C.-G. Ostenson, and G. Bluhm, "Aircraft noise and incidence of hypertension," *Epidemiology*, vol. 18, no. 6, pp. 716–721, Nov. 2007, doi: 10.1097/EDE.0b013e3181567e77.

[49] C. Eriksson, G. Bluhm, A. Hilding, C.-G. Ostenson, and G. Pershagen, "Aircraft noise and incidence of hypertension--gender specific effects," *Environ Res*, vol. 110, no. 8, pp. 764–772, Nov. 2010, doi: 10.1016/j.envres.2010.09.001.

[50] O. Hahad *et al.*, "Midregional pro atrial natriuretic peptide: a novel important biomarker for noise annoyance-induced cardiovascular morbidity and mortality?," *Clin Res Cardiol*, vol. 110, no. 1, pp. 29–39, Jan. 2021, doi: 10.1007/s00392-020-01645-6.

[51] P. Lercher, U. Widmann, and J. Thudium, "Hypotension and environmental noise: a replication study," *Int J Environ Res Public Health*, vol. 11, no. 9, pp. 8661–8688, Aug. 2014, doi: 10.3390/ijerph110908661.

[52] G. M. Aasvang *et al.*, "Burden of disease due to transportation noise in the Nordic countries," *Environ Res*, vol. 231, no. Pt 1, p. 116077, 2023, doi: 10.1016/j.envres.2023.116077.

[53] A. M. Abbasi, M. Motamedzade, M. Aliabadi, R. Golmohammadi, and L. Tapak, "Combined effects of noise and air temperature on human neurophysiological responses in a simulated indoor environment," *Appl Ergon*, vol. 88, p. 103189, 2020, doi: 10.1016/j.apergo.2020.103189.

[54] ACTRN12619000027112, "Assessing the health effects of six months of simulated wind farm infrasound: a community-based randomised controlled trial," 2019, [Online]. Available: https://www.cochranelibrary.com/central/doi/10.1002/central/CN-01946533/full

[55] S. Agarwal and B. L. Swami, "Road traffic noise, annoyance and community health survey - a case study for an Indian city," *Noise Health*, vol. 13, no. 53, pp. 272–6, 2011, doi: 10.4103/1463-1741.82959.

[56] I. Al-Harthy, P. Amoatey, A. Al-Mamun, Z. Alabri, and M. S. Baawain, "Assessment of noise levels and induced annoyance in nearby residential areas of an airport region in Oman," *Environ Sci Pollut Res Int*, vol. 28, no. 33, pp. 45596–45608, 2021, doi: 10.1007/s11356-021-13891-w.

[57] N. Z. Al-Mutairi, M. A. Al-Attar, and F. S. Al-Rukaibi, "Traffic-generated noise pollution: exposure of road users and populations in Metropolitan Kuwait," *Environ Monit Assess*, vol. 183, no. 1–4, pp. 65–75, 2011, doi: 10.1007/s10661-011-1906-0.

[58] E. Alkhalawi, E. Orban, S. Schramm, Z. Katsarava, B. Hoffmann, and S. Moebus, "Residential traffic noise exposure and headaches: Results from the population-based heinz nixdorf recall study.," *Noise & Health*, vol. 23, no. 108, pp. 1–10, 2021, doi: 10.4103/nah.NAH_1_20.

[59] P. Amoatey, H. Omidvarbona, M. S. Baawain, A. Al-Mayahi, A. Al-Mamun, and I. Al-Harthy, "Exposure assessment to road traffic noise levels and health effects in an arid urban area," *Environ Sci Pollut Res Int*, vol. 27, no. 28, pp. 35051–35064, 2020, doi: 10.1007/s11356-020-09785-y.
[60] P. Amoatey, I. Al-Harthy, M. A. Al-Mushaifari, K. Al-Jabri, and A. Al-Mamun, "Effect of ambient noise on indoor environments in a health care facility in Oman," *Environ Sci Pollut Res Int*, vol. 29, no. 10, pp. 15081–15097, 2022, doi: 10.1007/s11356-021-16875-y.
[61] W. Babisch, H. Ising, and J. E. Gallacher, "Health status as a potential effect modifier of the relation between noise annoyance and incidence of ischaemic heart disease," *Occup Environ Med*, vol. 60, no. 10, pp. 739–45, 2003, doi: 10.1136/oem.60.10.739.
[62] W. Babisch, B. Beule, M. Schust, N. Kersten, and H. Ising, "Traffic noise and risk of myocardial infarction," *Epidemiology*, vol. 16, no. 1, pp. 33–40, 2005, doi: 10.1097/01.ede.0000147104.84424.24.
[63] W. Babisch *et al.*, "Exposure modifiers of the relationships of transportation noise with high blood pressure and noise annoyance," *J Acoust Soc Am*, vol. 132, no. 6, pp. 3788–808, 2012, doi: 10.1121/1.4764881.
[64] W. Babisch, K. Wolf, and A. Peters, "Road traffic noise and hypertension - Results from the kora study," *Epidemiology*, vol. 23, no. 5, p. S607, 2012, doi: 10.1097/01.ede.0000417205.93172.49.
[65] C. Baudin, M. LefÈvre, P. Champelovier, J. Lambert, B. Laumon, and A. S. Evrard, "Self-rated health status in relation to aircraft noise exposure, noise annoyance or noise sensitivity: the results of a cross-sectional study in France," *BMC Public Health*, vol. 21, no. 1, p. 116, 2021, doi: 10.1186/s12889-020-10138-0.
[66] C. BAUDIN, M. LEFÈVRE, P. CHAMPELOVIER, J. LAMBERT, B. LAUMON, and A.-S. EVRARD, "Self-rated health status in relation to aircraft noise exposure, noise annoyance or noise sensitivity: the results of a cross-sectional study in France.," *BMC Public Health*, vol. 21, no. 1, pp. 1–11, 2021, doi: 10.1186/s12889-020-10138-0.
[67] G. Belojevic and M. Saric-Tanaskovic, "Prevalence of Arterial Hypertension and Myocardial Infarction in Relation to Subjective Ratings of Traffic Noise Exposure," *Noise Health*, vol. 4, no. 16, pp. 33–37, 2002.
[68] G. A. Belojević, B. D. Jakovljević, V. J. Stojanov, V. Z. Slepcević, and K. Z. Paunović, "Nighttime road-traffic noise and arterial hypertension in an urban population," *Hypertens Res*, vol. 31, no. 4, pp. 775–81, 2008, doi: 10.1291/hypres.31.775.
[69] G. Belojevic *et al.*, "Cardiovascular effects of environmental noise: research in Serbia," *Noise Health*, vol. 13, no. 52, pp. 217–20, 2011, doi: 10.4103/1463-1741.80156.
[70] M. E. Beutel *et al.*, "Noise Annoyance Is Associated with Depression and Anxiety in the General Population- The Contribution of Aircraft Noise," *PLoS One*, vol. 11, no. 5, p. e0155357, 2016, doi: 10.1371/journal.pone.0155357.
[71] A. Bosch *et al.*, "The Influence of annoyance due to aircraft noise on renal hemodynamic," *Nieren- und Hochdruckkrankheiten*, vol. 48, no. 11, p. 559, 2019, doi: 10.5414/NHX02069.
[72] A. Bosch *et al.*, "The influence of annoyance due to aircraft noise on renal hemodynamic," *Journal of Hypertension*, vol. 37, no. (Bosch A.; Kannenkeril D.; Ott C.; Harazny J.M.; Striepe K.; Karg M.V.; Schmieder R.E.) Department of Nephrology and Hypertension, Friedrich-Alexander-University Erlangen-Nürnberg (FAU), Erlangen, Germany(Harazny J.M.) Department of Pathophysiology, Unive, p. e200, 2019.
[73] A. Bosch *et al.*, "Annoyance due to noise triggers vascular changes in the renal hemodynamic," *Journal of Hypertension*, vol. 39, no. SUPPL 1, p. e169, 2021, doi: 10.1097/01.hjh.0000746280.75311.d6.
[74] A. Bosch *et al.*, "The influence of aircraft noise exposure on the systemic and renal haemodynamics," *European Journal of Preventive Cardiology*, vol. 29, no. 1, pp. 116–124, 2022, doi: 10.1093/eurjpc/zwaa036.
[75] E. V. Bräuner *et al.*, "Long-term wind turbine noise exposure and incidence of myocardial infarction in the Danish nurse cohort," *Environ Int*, vol. 121, no. Pt 1, pp. 794–802, 2018, doi: 10.1016/j.envint.2018.10.011.
[76] Byers JF, "Efficacy of a music intervention on noise annoyance and selected physiologic variables in cardiac surgery patients with high and low noise sensitivity.," University of Florida, 1996. [Online]. Available: https://search.ebscohost.com/login.aspx?direct=true&db=c8h&AN=109876914&site=ehost-live
[77] J. F. Byers and K. A. Smyth, "Effect of a music intervention on noise annoyance, heart rate, and blood pressure in cardiac surgery patients," *Am J Crit Care*, vol. 6, no. 3, pp. 183–91, 1997.
[78] M. Carugno *et al.*, "Effects of aircraft noise on annoyance, sleep disorders, and blood pressure among adult residents near the Orio al Serio International Airport (BGY), Italy," *Med Lav*, vol. 109, no. 4, pp. 253–263, 2018, doi: 10.23749/mdl.v109i4.7271.
[79] A. Chakraborty, A. Lahiri, U. Dasgupta, A. Saha, and S. K. Bhattacharya, "Cumulative Noise Exposure and Perceived Effects: A Comparative Study Among Different Occupational Groups in Kolkata," *Indian J Occup Environ Med*, vol. 26, no. 4, pp. 240–244, 2022, doi: 10.4103/ijoem.ijoem_51_22.

[80] F. A. M. Dias, W. T. Caiaffa, D. A. D. S. Costa, C. C. Xavier, F. A. Proietti, and A. A. L. Friche, “Noise annoyance, sociodemographic and health patterns, and neighborhood perceptions in a brazilian metropolis: Bh health study,” *Revista Brasileira de Epidemiologia*, vol. 24, no. (Dias F.A.M., fernandaabalen@gmail.com; Caiaffa W.T.; Friche A.A.L.) School of Medicine, Universidade Federal de Minas Gerais, Belo Horizonte (MG), Brazil(Dias F.A.M., fernandaabalen@gmail.com; Caiaffa W.T.; Costa D.A.D.S.; Xavier C.C.; Proietti F.A.; Fri, 2021, doi: 10.1590/1980-549720210038.

[81] J. Dopico *et al.*, “How Do Road Traffic Noise and Residential Greenness Correlate with Noise Annoyance and Long-Term Stress? Protocol and Pilot Study for a Large Field Survey with a Cross-Sectional Design,” *Int J Environ Res Public Health*, vol. 20, no. 4, 2023, doi: 10.3390/ijerph20043203.

[82] DRKS00011362, “Fragile sleep under Aircraft Noise and Circadian Shift - Examination about the influence of air traffic noise on the vulnerable sleep induced by shift of circadian rhythm,” 2016, [Online]. Available: https://www.cochranelibrary.com/central/doi/10.1002/central/CN-01858251/full

[83] A. M. Dzhambov *et al.*, “Association between community noise and adiposity in patients with cardiovascular disease,” *Noise Health*, vol. 19, no. 91, pp. 270–277, 2017, doi: 10.4103/nah.NAH_78_16.

[84] C. Eriksson, M. E. Nilsson, S. M. Willers, L. Gidhagen, T. Bellander, and G. Pershagen, “Traffic noise and cardiovascular health in Sweden: the roadside study,” *Noise Health*, vol. 14, no. 59, pp. 140–7, 2012, doi: 10.4103/1463-1741.99864.

[85] C. Eriksson, T. Bodin, and J. Selander, “Burden of disease from road traffic and railway noise – a quantification of healthy life years lost in Sweden,” *Scandinavian Journal of Work, Environment and Health, Supplement*, vol. 43, no. 6, pp. 519–525, 2017, doi: 10.5271/sjweh.3653.

[86] I. C. Eze *et al.*, “Transportation noise exposure, noise annoyance and respiratory health in adults: A repeated-measures study,” *Environ Int*, vol. 121, no. Pt 1, pp. 741–750, 2018, doi: 10.1016/j.envint.2018.10.006.

[87] I. C. Eze *et al.*, “Incidence of depression in relation to transportation noise exposure and noise annoyance in the SAPALDIA study,” *Environ Int*, vol. 144, p. 106014, 2020, doi: 10.1016/j.envint.2020.106014.

[88] Z. U. R. Farooqi *et al.*, “Assessment of noise pollution and its effects on human health in industrial hub of Pakistan,” *Environ Sci Pollut Res Int*, vol. 27, no. 3, pp. 2819–2828, 2020, doi: 10.1007/s11356-019-07105-7.

[89] Z. U. R. Farooqi, I. Ahmad, N. Zeeshan, P. Ilić, M. Imran, and M. F. Saeed, “Urban noise assessment and its nonauditory health effects on the residents of Chiniot and Jhang, Punjab, Pakistan,” *Environ Sci Pollut Res Int*, vol. 28, no. 39, pp. 54909–54921, 2021, doi: 10.1007/s11356-021-14340-4.

[90] E. Flanagan, E. Malmqvist, A. Oudin, K. Sunde Persson, J. Alkan Ohlsson, and K. Mattisson, “Health impact assessment of road traffic noise exposure based on different densification scenarios in Malmö, Sweden,” *Environ Int*, vol. 174, p. 107867, 2023, doi: 10.1016/j.envint.2023.107867.

[91] M. M. Fooladi, “Involuntary and persistent environmental noise influences health and hearing in Beirut, Lebanon,” *Journal of Environmental and Public Health*, vol. 2012, no. (Fooladi M.M., fooladi@hotmail.com) Council for International Exchange of Scholars, A Division of the Institute of International Education, 3007 Tilden Street NW, Washington, DC 20008, United States(Fooladi M.M., fooladi@hotmail.com) College of Nursing, F, 2012, doi: 10.1155/2012/235618.

[92] M. Foraster *et al.*, “Impact of long- and shorter-term annoyance to transportation noise at home on physical inactivity,” *Tropical Medicine and International Health*, vol. 20, no. (Foraster M.; Eze I.; Vienneau D.; Héritier H.; Röösli M.; Probst-Hensch N.) Swiss Tropical and Public Health Institute, Basel, Switzerland(Foraster M.; Eze I.; Vienneau D.; Héritier H.; Röösli M.; Probst-Hensch N.) University of Basel, Basel, Switzerland, p. 265, 2015, doi: 10.1111/tmi.12574.

[93] A. Fyhri and G. M. Aasvang, “Noise, sleep and poor health: Modeling the relationship between road traffic noise and cardiovascular problems,” *Science of the Total Environment*, vol. 408, no. 21, pp. 4935–4942, 2010, doi: 10.1016/j.scitotenv.2010.06.057.

[94] M. M. Haines, S. A. Stansfeld, R. F. Job, B. Berglund, and J. Head, “Chronic aircraft noise exposure, stress responses, mental health and cognitive performance in school children,” *Psychol Med*, vol. 31, no. 2, pp. 265–77, 2001, doi: 10.1017/s0033291701003282.

[95] D. Hauptvogel, D. Schreckenberg, T. Rothmund, M.-T. Schmitz, and S. Bartels, “Being a Fair Neighbor—Towards a Psychometric Inventory to Assess Fairness-Related Perceptions of Airports by Residents—Development and Validation of the Aircraft Noise-Related Fairness Inventory (fAIR-In),” *International Journal of Environmental Research and Public Health*, vol. 20, no. 12, 2023, doi: 10.3390/ijerph20126113.

[96] IRCT201411304443N16, “music therapy and mechanical ventilation,” 2015, [Online]. Available: https://www.cochranelibrary.com/central/doi/10.1002/central/CN-01800045/full

[97] H. Ising, E. Rebentisch, F. Poustka, and I. Curio, “Annoyance and health risk caused by military low-altitude flight noise,” *Int Arch Occup Environ Health*, vol. 62, no. 5, pp. 357–63, 1990, doi: 10.1007/bf00381365.

[98] H. Ising, H. Lange-Asschenfeldt, H. J. Moriske, J. Born, and M. Eilts, “Low frequency noise and stress: bronchitis and cortisol in children exposed chronically to traffic noise and exhaust fumes,” *Noise Health*, vol. 6, no. 23, pp. 21–8, 2004.

[99] N. Itzkowitz *et al.*, “Aircraft noise and cardiovascular morbidity and mortality near Heathrow Airport: A case-crossover study,” *Environ Int*, vol. 177, p. 108016, 2023, doi: 10.1016/j.envint.2023.108016.

[100] L. Jarup *et al.*, “Hypertension and Exposure to Noise near Airports (HYENA): study design and noise exposure assessment,” *Environ Health Perspect*, vol. 113, no. 11, pp. 1473–8, 2005, doi: 10.1289/ehp.8037.

[101] H. A. R. Jensen, B. Rasmussen, and O. Ekholm, “Neighbour and traffic noise annoyance: a nationwide study of associated mental health and perceived stress,” *Eur J Public Health*, vol. 28, no. 6, pp. 1050–1055, 2018, doi: 10.1093/eurpub/cky091.

[102] H. A. R. Jensen, B. Rasmussen, and O. Ekholm, “Neighbour noise annoyance is associated with various mental and physical health symptoms: results from a nationwide study among individuals living in multi-storey housing,” *BMC Public Health*, vol. 19, no. 1, p. 1508, 2019, doi: 10.1186/s12889-019-7893-8.

[103] A. Kasicka-Jonderko *et al.*, “Extracirculatory effects of noise of various frequency spectra in humans--effect of pink and blue noise on gastric myoelectrical activity and gastrointestinal passage of nutrients,” *J Smooth Muscle Res*, vol. 43, no. 1, pp. 25–42, 2007, doi: 10.1540/jsmr.43.25.

[104] Z. Kooshanfar *et al.*, “Sources of noise and their effects on nurses in intensive care units: A cross sectional study,” *International Journal of Africa Nursing Sciences*, vol. 16, no. (Kooshanfar Z.) Nursing (MSN), School of Nursing and Midwifery, Guilan University of Medical Sciences, Rasht, Iran(Ashrafi S., sadra.ashrafi@sbmu.ac.ir) Student Research Committee, Chronic Kidney Disease Research Center(CKDRC), Shahid Beheshti University, 2022, doi: 10.1016/j.ijans.2022.100403.

[105] S. Lamb and K. C. S. Kwok, “A longitudinal investigation of work environment stressors on the performance and wellbeing of office workers.,” *Applied Ergonomics*, vol. 52, pp. 104–111, 2016, doi: 10.1016/j.apergo.2015.07.010.

[106] S. L. Lusk, B. M. Hagerty, B. Gillespie, and C. C. Caruso, “Chronic effects of workplace noise on blood pressure and heart rate,” *Archives of Environmental Health*, vol. 57, no. 4, pp. 273–281, 2002, doi: 10.1080/00039890209601410.

[107] T. Mabote *et al.*, “Effect of music and noise on haemodynamics in patients with heart failure. HeartCycle (FP7-216695) European union 7th framework programme,” *European Journal of Heart Failure, Supplement*, vol. 11, no. (Mabote T.; Torabi A.; Antony R.; Zhang Z.; Pellicori P.; Costanzo P.; Hobkirk J.; Clark A.L.; Cleland J.G.F.) University of Hull, Department of Academic Cardiology, Hull, United Kingdom, p. S35, 2012, doi: 10.1093/eurjhf/hss006.

[108] V. Malinauskiene and A. Azaraviciene, “The associations between urban traffic emissions on myocardial infarction risk,” *Epidemiology*, vol. 22, no. (Malinauskiene V.; Azaraviciene A.) Institute of Cardiology, Kaunas University of Medicine, Kaunas, Lithuania, p. S256, 2011, doi: 10.1097/01.ede.0000392480.85474.57.

[109] Marshall Nathaniel S. *et al.*, “The Health Effects of 72 Hours of Simulated Wind Turbine Infrasound: A Double-Blind Randomized Crossover Study in Noise-Sensitive, Healthy Adults,” *Environmental Health Perspectives*, vol. 131, no. 3, p. 037012, doi: 10.1289/EHP10757.

[110] S. Melamed, J. Luz, and M. S. Green, “Noise exposure, noise annoyance and their relation to psychological distress, accident and sickness absence among blue-collar workers--the Cordis Study.,” *Israel journal of medical sciences*, vol. 28, no. 8–9, pp. 629–635, 1992.

[111] J. Méline, A. Van Hulst, F. Thomas, N. Karusisi, and B. Chaix, “Transportation noise and annoyance related to road traffic in the French RECORD study,” *Int J Health Geogr*, vol. 12, p. 44, 2013, doi: 10.1186/1476-072x-12-44.

[112] H. Moller, “Physiological and psychological effects of infrasound on humans,” *Journal of Low Frequency Noise and Vibration*, vol. 3, no. 1, pp. 1–16, 1984, doi: 10.1177/026309238400300101.

[113] W. E. Morrison, E. C. Haas, D. H. Shaffner, E. S. Garrett, and J. C. Fackler, “Noise, stress, and annoyance in a pediatric intensive care unit,” *Crit Care Med*, vol. 31, no. 1, pp. 113–9, 2003, doi: 10.1097/00003246-200301000-00018.

[114] NCT04809545, "Effect of Soundscape on People With Dementia," 2021, [Online]. Available: https://www.cochranelibrary.com/central/doi/10.1002/central/CN-02253076/full
[115] NCT05319262, "Metabolic and Cognitive Consequences of Noise-induced Sleep Disturbance," 2022, [Online]. Available: https://www.cochranelibrary.com/central/doi/10.1002/central/CN-02392320/full
[116] NCT05611619, "Low Particle Emission and Low Noise Tyres," 2022, [Online]. Available: https://www.cochranelibrary.com/central/doi/10.1002/central/CN-02495322/full
[117] T. L. Nguyen, B. L. Trieu, Y. Hiraguri, M. Morinaga, T. Morihara, and T. Yano, "Effects of Changes in Acoustic and Non-Acoustic Factors on Public Health and Reactions: Follow-Up Surveys in the Vicinity of the Hanoi Noi Bai International Airport," *Int J Environ Res Public Health*, vol. 17, no. 7, 2020, doi: 10.3390/ijerph17072597.
[118] M. Nitschke, G. Tucker, D. L. Simon, A. L. Hansen, and D. L. Pisaniello, "The link between noise perception and quality of life in South Australia," *Noise Health*, vol. 16, no. 70, pp. 137–42, 2014, doi: 10.4103/1463-1741.134913.
[119] E. O. Okokon, A. W. Turunen, S. Ung-Lanki, A.-K. Vartiainen, P. Tiittanen, and T. Lanki, "Road-traffic noise: Annoyance, risk perception, and noise sensitivity in the finnish adult population," *International Journal of Environmental Research and Public Health*, vol. 12, no. 6, pp. 5712–5734, 2015, doi: 10.3390/ijerph120605712.
[120] E. O. Okokon, T. Yli-Tuomi, A. W. Turunen, P. Tiittanen, J. Juutilainen, and T. Lanki, "Traffic noise, noise annoyance and psychotropic medication use," *Environ Int*, vol. 119, pp. 287–294, 2018, doi: 10.1016/j.envint.2018.06.034.
[121] D. Ouis, "Annoyance caused by exposure to road traffic noise: An update," *Noise and Health*, vol. 4, no. 15, pp. 69–72, 2002.
[122] M. Pawlaczyk-Łuszczyńska, K. Zaborowski, A. Dudarewicz, M. Zamojska-Daniszewska, and M. Waszkowska, "Response to noise emitted by wind farms in people living in nearby areas," *International Journal of Environmental Research and Public Health*, vol. 15, no. 8, 2018, doi: 10.3390/ijerph15081575.
[123] A. H. Poulsen *et al.*, "Short-term nighttime wind turbine noise and cardiovascular events: A nationwide case-crossover study from Denmark," *Environ Int*, vol. 114, pp. 160–166, 2018, doi: 10.1016/j.envint.2018.02.030.
[124] A. H. Poulsen *et al.*, "Long-term exposure to wind turbine noise and redemption of antihypertensive medication: A nationwide cohort study," *Environ Int*, vol. 121, no. Pt 1, pp. 207–215, 2018, doi: 10.1016/j.envint.2018.08.054.
[125] A. H. Poulsen *et al.*, "Long-Term Exposure to Wind Turbine Noise and Risk for Myocardial Infarction and Stroke: A Nationwide Cohort Study," *Environ Health Perspect*, vol. 127, no. 3, p. 37004, 2019, doi: 10.1289/ehp3340.
[126] M. P. J. Pulles, W. Biesiot, and R. Stewart, "Adverse effects of environmental noise on health: An interdisciplinary approach," *Environment International*, vol. 16, no. 4–6, pp. 437–445, 1990, doi: 10.1016/0160-4120(90)90012-U.
[127] J. Radun, H. Maula, V. Rajala, M. Scheinin, and V. Hongisto, "Speech is special: The stress effects of speech, noise, and silence during tasks requiring concentration," *Indoor Air*, vol. 31, no. 1, pp. 264–274, 2021, doi: 10.1111/ina.12733.
[128] M. M. Rahman *et al.*, "Perceived Noise Pollution and Self-Reported Health Status among Adult Population of Bangladesh," *International Journal of Environmental Research and Public Health*, vol. 19, no. 4, 2022, doi: 10.3390/ijerph19042394.
[129] N. Riedel *et al.*, "Home as a Place of Noise Control for the Elderly? A Cross-Sectional Study on Potential Mediating Effects and Associations between Road Traffic Noise Exposure, Access to a Quiet Side, Dwelling-Related Green and Noise Annoyance," *Int J Environ Res Public Health*, vol. 15, no. 5, 2018, doi: 10.3390/ijerph15051036.
[130] N. Riedel *et al.*, "Urban road traffic noise and noise annoyance-a study on perceived noise control and its value among the elderly," *Eur J Public Health*, vol. 29, no. 2, pp. 377–379, 2019, doi: 10.1093/eurpub/cky141.
[131] M. Rojek, M. Rajzer, W. Wojciechowska, P. Skalski, T. Pizon, and D. Czarnecka, "Nighttime aircraft noise effect on blood pressure profile," *European Heart Journal*, vol. 39, no. (Rojek M.; Rajzer M.; Wojciechowska W.; Pizon T.; Czarnecka D.) Jagiellonian University Medical College, 1st Department of Cardiology,Interventional Electrocardiology and Arterial Hypertension, Krakow, Poland(Skalski P.) Institute of Aviation, Warsaw, Pol, pp. 497–498, 2018, doi: 10.1093/eurheartj/ehy565.P2542.

[132] M. Rojek, M. Rajzer, W. Wojciechowska, P. Skalski, T. Pizon, and D. Czarnecka, "Environmental noise and arterial stiffness in the context of hypertension," *European Journal of Preventive Cardiology*, vol. 26, no. (Rojek M.; Rajzer M.; Wojciechowska W.; Czarnecka D.) Jagiellonian University Medical College, 1st Department of Cardiology, Interventional Electrocardiology and Arterial Hypertension, Krakow, Poland(Skalski P.) Institute of Aviation, Warsaw, Poland(Pizon, pp. S75–S76, 2019, doi: 10.1177/2047487319860053.

[133] M. Rojek, W. Wojciechowska, A. Januszewicz, D. Czarnecka, P. Skalski, and M. Rajzer, "The relation of nocturnal exposure to aircraft noise and aircraft noise–induced insomnia with blood pressure," *Polish Archives of Internal Medicine*, vol. 131, no. 1, pp. 33–41, 2021, doi: 10.20452/pamw.15716.

[134] S. Sandrock, M. Schutte, and B. Griefahn, "Mental strain and annoyance during cognitive performance in different traffic noise conditions.," *Ergonomics*, vol. 53, no. 8, pp. 962–971, 2010.

[135] F. Schmidt *et al.*, "Nighttime aircraft noise impairs endothelial function and increases blood pressure in patients with or at high risk for coronary artery disease," *Clinical Research in Cardiology*, no. (Schmidt F.; Kolle K.; Kreuder K.; Schnorbus B.; Wild P.; Gori T.; Münzel T., tmuenzel@uni-mainz.de) 2 Medical Clinic, Cardiology, University Medical Center Mainz, Johannes Gutenberg University, Langenbeckstrasse 1, Mainz, 55131, Germany(Hechtner M.; Bind, 2014, doi: 10.1007/s00392-014-0751-x.

[136] F. Schmidt *et al.*, "Nighttime aircraft noise impairs endothelial function and increases blood pressure in patients with or at high risk for coronary artery disease," *Clin Res Cardiol*, vol. 104, no. 1, pp. 23–30, 2015, doi: 10.1007/s00392-014-0751-x.

[137] F. P. Schmidt *et al.*, "The impact of aircraft noise on vascular and cardiac function in relation to noise event number: a randomized trial," *Cardiovasc Res*, vol. 117, no. 5, pp. 1382–1390, 2021, doi: 10.1093/cvr/cvaa204.

[138] D. Schreckenberg, M. Meis, C. Kahl, C. Peschel, and T. Eikmann, "Aircraft noise and quality of life around Frankfurt Airport," *Int J Environ Res Public Health*, vol. 7, no. 9, pp. 3382–405, 2010, doi: 10.3390/ijerph7093382.

[139] D. Schreckenberg, B. Griefahn, and M. Meis, "The associations between noise sensitivity, reported physical and mental health, perceived environmental quality, and noise annoyance.," *Noise & health*, vol. 12, no. 46, pp. 7–16, 2010, doi: 10.4103/1463-1741.59995.

[140] J. Selander *et al.*, "Long-term exposure to road traffic noise and myocardial infarction," *Epidemiology*, vol. 20, no. 2, pp. 272–9, 2009, doi: 10.1097/EDE.0b013e31819463bd.

[141] A. Şentop Dümen and K. Şaher, "Noise annoyance during COVID-19 lockdown: A research of public opinion before and during the pandemic," *J Acoust Soc Am*, vol. 148, no. 6, p. 3489, 2020, doi: 10.1121/10.0002667.

[142] M. Smith, S. Rocha, M. Witte, and M. Basner, "Self-reported sleep disturbance by aircraft noise around Atlanta airport," *Sleep*, vol. 42, no. (Smith M.; Rocha S.; Witte M.; Basner M.) Division of Sleep and Chronobiology, University of Pennsylvania, Philadelphia, PA, United States, p. A54, 2019, doi: 10.1093/sleep/zsz067.131.

[143] L. Sobotova, J. Jurkovicova, Z. Stefanikova, L. Sevcikova, and L. Aghova, "Community response to environmental noise and the impact on cardiovascular risk score," *Sci Total Environ*, vol. 408, no. 6, pp. 1264–70, 2010, doi: 10.1016/j.scitotenv.2009.12.033.

[144] R. M. Stamatin and C. Petrea, "The impact of high noise exposure on textile workers health-a retrospective 3 years study in a textile factory," *Occupational and Environmental Medicine*, vol. 75, no. (Stamatin R.M.) "A. Popescu" Emergency Military Hospital, Focşani, Romania(Petrea C.) National Sanitary Veterinary Authority-Vrancea County, Focsani, Romania, pp. A536–A537, 2018, doi: 10.1136/oemed-2018-ICOHabstracts.1521.

[145] S. A. Stansfeld and M. Shipley, "Noise sensitivity and future risk of illness and mortality," *Science of the Total Environment*, vol. 520, no. (Stansfeld S.A., s.a.stansfeld@qmul.ac.uk) Centre for Psychiatry, Wolfson Institute of Preventive Medicine, Barts and the London School of Medicine and Dentistry,Queen Mary University of London, Charterhouse Square, London, United Kingdom(Shipley M., mart, pp. 114–119, 2015, doi: 10.1016/j.scitotenv.2015.03.053.

[146] S. Stansfeld, C. Clark, M. Smuk, J. Gallacher, and W. Babisch, "Road traffic noise, noise sensitivity, noise annoyance, psychological and physical health and mortality," *Environ Health*, vol. 20, no. 1, p. 32, 2021, doi: 10.1186/s12940-021-00720-3.

[147] L. Thiesse *et al.*, "Transportation noise impairs cardiovascular function without altering sleep: The importance of autonomic arousals," *Environ Res*, vol. 182, p. 109086, 2020, doi: 10.1016/j.envres.2019.109086.

[148] M. Tobollik, M. Hintzsche, J. Wothge, T. Myck, and D. Plass, "Burden of Disease Due to Traffic Noise in Germany," *Int J Environ Res Public Health*, vol. 16, no. 13, 2019, doi: 10.3390/ijerph16132304.

[149] K. H. Tsoi, B. P. Y. Loo, X. Li, and K. Zhang, "The co-benefits of electric mobility in reducing traffic noise and chemical air pollution: Insights from a transit-oriented city," *Environ Int*, vol. 178, p. 108116, 2023, doi: 10.1016/j.envint.2023.108116.
[150] A. W. Turunen, P. Tiittanen, T. Yli-Tuomi, P. Taimisto, and T. Lanki, "Self-reported health in the vicinity of five wind power production areas in Finland," *Environ Int*, vol. 151, p. 106419, 2021, doi: 10.1016/j.envint.2021.106419.
[151] F. J. van Dijk, J. H. Verbeek, and F. F. de Fries, "Non-auditory effects of noise in industry. V. A field study in a shipyard," *Int Arch Occup Environ Health*, vol. 59, no. 1, pp. 55–62, 1987, doi: 10.1007/bf00377679.
[152] F. J. H. Van Dijk, A. M. Souman, and F. F. De Vries, "Non-auditory effects of noise in industry. VI. A final study in industry," *International Archives of Occupational and Environmental Health*, vol. 59, no. 2, pp. 133–145, 1987, doi: 10.1007/BF00378491.
[153] A. E. Wallas *et al.*, "Noise exposure and childhood asthma up to adolescence," *Environ Res*, vol. 185, p. 109404, 2020, doi: 10.1016/j.envres.2020.109404.
[154] P. WALLNER, H.-P. HUTTER, and H. MOSHAMMER, "Evaluating an 80 Hz tonal noise from a hydropower plant.," *International Journal of Occupational Medicine & Environmental Health*, vol. 32, no. 3, pp. 401–411, 2019, doi: 10.13075/ijomeh.1896.01361.
[155] W. Wang *et al.*, "Associations between personal noise exposure and heart rate variability were modified by obesity and PM(2.5): The study among obese and normal-weight adults (SONA)," *Environ Res*, vol. 214, no. Pt 2, p. 113888, 2022, doi: 10.1016/j.envres.2022.113888.
[156] K. P. Waye, J. Bengtsson, R. Rylander, F. Hucklebridge, P. Evans, and A. Clow, "Low frequency noise enhances cortisol among noise sensitive subjects during work performance," *Life Sciences*, vol. 70, no. 7, pp. 745–758, 2002, doi: 10.1016/S0024-3205(01)01450-3.
[157] Webb JJ, "Effects of hospital noise on heart rate variability of acutely ill adults.," University of Tennessee Center for the Health Sciences, 1998. [Online]. Available: https://search.ebscohost.com/login.aspx?direct=true&db=c8h&AN=109878302&site=ehost-live
[158] S. N. Willich, K. Wegscheider, M. Stallmann, and T. Keil, "Noise burden and the risk of myocardial infarction," *Eur Heart J*, vol. 27, no. 3, pp. 276–82, 2006, doi: 10.1093/eurheartj/ehi658.
[159] W. Wojciechowska *et al.*, "Impact of reduction in aircraft noise exposure on blood pressure and arterial stiffness during COVID-19 pandemic: Cross-sectional and longitudinal study," *Journal of Hypertension*, vol. 39, no. SUPPL 1, p. e204, 2021, doi: 10.1097/01.hjh.0000746748.93286.f4.
[160] W. Wojciechowska *et al.*, "Blood Pressure and Arterial Stiffness in Association With Aircraft Noise Exposure:Long-Term Observation and Potential Effect of COVID-19 Lockdown," *Hypertension*, vol. 79, no. 2, pp. 325–334, 2022, doi: 10.1161/hypertensionaha.121.17704.
[161] W. Wojciechowska, "NOISE POLLUTION AND ARTERIAL HYPERTENSION," *Journal of Hypertension*, vol. 41, no. (Wojciechowska W.) 1st Department of Cardiology,Interventional Electrocardiology and Arterial Hypertension, Jagiellonian University Medical College, Poland, p. e147, 2023.
[162] J. Wothge, C. Belke, U. Möhler, R. Guski, and D. Schreckenberg, "The Combined Effects of Aircraft and Road Traffic Noise and Aircraft and Railway Noise on Noise Annoyance-An Analysis in the Context of the Joint Research Initiative NORAH," *Int J Environ Res Public Health*, vol. 14, no. 8, 2017, doi: 10.3390/ijerph14080871.
[163] W. T. Yang *et al.*, "Road Traffic Noise, Air Pollutants, and the Prevalence of Cardiovascular Disease in Taichung, Taiwan," *Int J Environ Res Public Health*, vol. 15, no. 8, 2018, doi: 10.3390/ijerph15081707.
[164] W. L. Zijlema, D. W. Morley, R. P. Stolk, and J. G. Rosmalen, "Noise and somatic symptoms: A role for personality traits?," *International journal of hygiene and environmental health*, vol. 218, no. 6, pp. 543–549, 2015, doi: 10.1016/j.ijheh.2015.05.001.
